# Titanium dioxide nanoparticles impair the inner blood-retinal barrier and retinal electrophysiology through rapid ADAM17 activation and claudin-5 degradation

**DOI:** 10.1186/s12989-020-00395-7

**Published:** 2021-01-09

**Authors:** Yen-Ju Chan, Po-Lin Liao, Chi-Hao Tsai, Yu-Wen Cheng, Fan-Li Lin, Jau-Der Ho, Ching-Yi Chen, Ching-Hao Li

**Affiliations:** 1 Graduate Institute of Medical Sciences, College of Medicine, Taipei Medical University, Taipei, Taiwan; 2 Department of Physiology, School of Medicine, College of Medicine, Taipei Medical University, 250 Wuxing Street, Taipei, 110 Taiwan; 3 School of Pharmacy, Taipei Medical University, Taipei, Taiwan; 4 Institute of Food Safety and Health Risk Assessment, School of Pharmaceutical Sciences, National Yang-Ming University, Taipei, Taiwan; 5 Menzies Institute for Medical Research, University of Tasmania, Hobart, Tasmania Australia; 6 Department of Ophthalmology, Taipei Medical University, Taipei, Taiwan

**Keywords:** Titanium dioxide nanoparticles, Endothelial cells, Claudin-5, ADAM17, Blood-retinal barrier

## Abstract

**Background:**

Depending on their distinct properties, titanium dioxide nanoparticles (TiO_2_-NPs) are manufactured extensively and widely present in our daily necessities, with growing environmental release and public concerns. In sunscreen formulations, supplementation of TiO_2_-NPs may reach up to 25% (w/w). Ocular contact with TiO_2_-NPs may occur accidentally in certain cases, allowing undesirable risks to human vision. This study aimed to understand the barrier integrity of retinal endothelial cells in response to TiO_2_-NP exposure. bEnd.3 cells and human retinal endothelial cells (HRECs) were exposed to TiO_2_-NP, followed by examination of their tight junction components and functions.

**Results:**

TiO2-NP treatment apparently induced a broken structure of the junctional plaques, conferring decreased transendothelial electrical resistance, a permeable paracellular cleft, and improved cell migration in vitro. This might involve rapid activation of metalloproteinase, a disintegrin and metalloproteinase 17 (ADAM17), and ADAM17-mediated claudin-5 degradation. For the in vivo study, C57BL/6 mice were administered a single dose of TiO2-NP intravitreally and then subjected to a complete ophthalmology examination. Fluorescein leakage and reduced blood flow at the optical disc indicated a damaged inner blood-retinal barrier induced by TiO_2_-NPs. Inappreciable change in the thickness of retinal sublayers and alleviated electroretinography amplitude were observed in the TiO_2_-NP-treated eyes.

**Conclusions:**

Overall, our data demonstrate that TiO2-NP can damage endothelial cell function, thereby affecting retinal electrophysiology.

**Supplementary Information:**

The online version contains supplementary material available at 10.1186/s12989-020-00395-7.

## Background

Titanium dioxide (TiO_2_), the naturally occurring oxide of titanium, is abundant in the earth’s crust. It is frequently incorporated in many daily necessities (e.g., toothpaste, food, medicine, and cosmetics) as a white colorant. TiO_2_ has excellent surface activity to ultraviolet (UV) radiation, either by scattering sunlight or by absorbing the spectrum (a semiconducting effect). Therefore, TiO_2_ has been approved as a mineral UV filter at a maximum supplementation of 25% in sunscreen products. However, sunscreens containing TiO_2_ macroparticles are often difficult to apply and leave a chalky appearance. These unfavorable disadvantages could be mitigated by shrinking the TiO_2_ particle size to the nano range. Thus, TiO_2_ nanoparticles (TiO_2_-NPs) are more commonly used in sunscreen formulations [1, 2]. The explosive expansion in the application of TiO_2_-NPs has thus led to their safety being highlighted and questioned.

Nanoparticles are suspected to enter the human body easily through 3 major routes: inhalation, ingestion, and dermal deposition. An acute injury to the lungs was found to correspond to TiO_2_-NP inhalation [3, 4]. Due to its pulmonary toxicity and the Group 2B classification from IARC, the use of TiO_2_-NP in sprays is not recommended [5]. Upon dermal exposure, either in healthy or damaged skin, the distribution of TiO_2_-NP is confined to the outer layers of the stratum corneum [6]; whereas in another study, TiO_2_-NPs were found to penetrate the epidermis slightly, but did not permeate the dermis or subcutis [7]. Owing to the limited penetration of TiO_2_-NP into circulation, the Scientific Committee on Consumer Safety (SCCS) deems that TiO_2_-NP does not present any health issues upon dermal exposure [5]. However, this has been disputed in recent years. For example, the human eye might incidentally come in contact with TiO_2_-NPs; however, information regarding ocular hazards is limited. In vitro exposure of TiO_2_-NPs (> 5 μg/mL) has been reported to lower cell viability of retinal pigment epithelial (RPE) cells and human lens epithelial cells [8–10]. In combination with UV treatment, the TiO_2_-NPs were found to be capable of generating reactive oxygen species (ROS), resulting in apparent phototoxic damage [8–10]. Mice which received repetitive topical administration of TiO_2_-NP (400–1000 ng/eye) displayed goblet cell exhaustion, decreased tear mucin secretion, corneal haze, and inflammation on the ocular surface [11, 12]. Intravitreal (ITV) administration of TiO_2_-NPs (130 ng/mL) did not alter the histologic integrity of the retina, but effectively repressed angiogenesis [13], suggesting that retinal endothelial cells might be the target of TiO_2_-NP challenge.

The human eye works much like a digital camera. Light enters the cornea, passes through the pupil and lens, and falls on the retina. Light-sensitive photoreceptors (rods and cones), localized in the outer retina, convert photons into electrical impulses. To maintain retinal homeostasis and visual function, a restricted interface of permeability exists between the blood and retina, which is termed the blood-retinal barrier (BRB) [14]. The BRB is comparable to the blood brain barrier and regulates ion, protein, and water transport across retinal capillaries. The structure of the BRB comprises two distinct levels: the inner BRB and the outer BRB, which consist of retinal capillary endothelial cells and RPECs, respectively. The former primarily regulates transport across retinal microvessels, and the latter regulates the movement of solutes and nutrients between the choroid and sub-retinal space. Loss of the BRB is a crucial determinant of several retinal diseases, including macular edema, thickening of retinal tissue, diabetic retinopathy, and age-related macular degeneration [15, 16]. In addition, pro-inflammatory cytokines, such as tumor necrosis factor-α (TNF-α), play a causative role in BRB disruption [17].

The main ultrastructural levels of the inner and outer BRB are formed by tight junctions (TJs). The constituents of the TJ are complex and involve more than 40 different proteins. Among these factors, claudins are important for cleft sealing [18, 19], eventually defining the integrity and permeable selectivity of TJs. Claudins are small transmembrane proteins that span the cell membrane four times. Their carboxy-terminal end contains a PDZ-binding motif, which is necessary for pairing with zonula occludens-1 (ZO-1), a TJ scaffolding protein [18–20]. In mammals, the 27 claudin isoforms are highly conserved in their protein secondary structure [19], but are differentially expressed among tissues. Knockdown of barrier-forming claudins (e.g., claudin-1 or claudin-5) dramatically increases paracellular permeability. Conversely, an abundance of TJ plaque was found upon overexpression of these claudins [19, 21]. In addition, non-claudin members (e.g., occludin and junctional adhesion molecules) appear in TJ plaques, contributing to the physiological barrier function. Moreover, adherens junctions have also been recognized to facilitate intercellular adhesion and TJ formation [20]. This study aimed to characterize the effects of TiO_2_-NP on retinal function, especially on RPEC- and endothelial cell-derived barrier permeability.

## Results

### Characteristics of TiO2 particles

The shape, size, and size distribution of the TiO_2_ particles are summarized in Table S1. TEM imaging showed that the primary TiO_2_-NPs contained an anatase core and an elliptical shape with an average diameter of 42 ± 3 nm. TiO_2_-MPs morphologically appeared as compacted crystals with diameters > 1 μm. The hydrodynamic diameter of the TiO_2_-NPs was 184.7 ± 64.1 nm. The average ζ-potential and absorbance spectra also showed a difference between the TiO_2_-NPs and TiO_2_-MPs.

### TiO2-NP treatment mildly reduced the cell density of bEnd.3 endothelial cells and RPECs

Treatment of bEnd.3 cells with serially diluted TiO_2_-NPs (or TiO_2_-MPs) for 24 h revealed a mild reduction in cell density (% related to control) as determined by an MTT assay. At 1000 ng/mL of TiO_2_ particles, the vital bEnd.3 cells were 83.8 ± 2.1% (TiO_2_-NPs) and 92.8 ± 3.7% (TiO_2_-MPs), respectively. No morphological distinctions were identified among the test groups, suggesting that a slight proliferation inhibition rather than cytotoxicity might be induced by TiO_2_ particles. Similar results were demonstrated for RPECs. Overall, the in vitro cytotoxicity of TiO_2_-NPs at < 1000 ng/mL is limited in bEnd.3 cells and RPECs. However, TiO_2_-NP reduced the viability of primary human retinal endothelial cells (HRECs) (Table S2).

### TiO2-NP reduced endothelial claudin-5 protein levels without affecting claudin-1 expression in treated RPECs

**Fig. 1:**
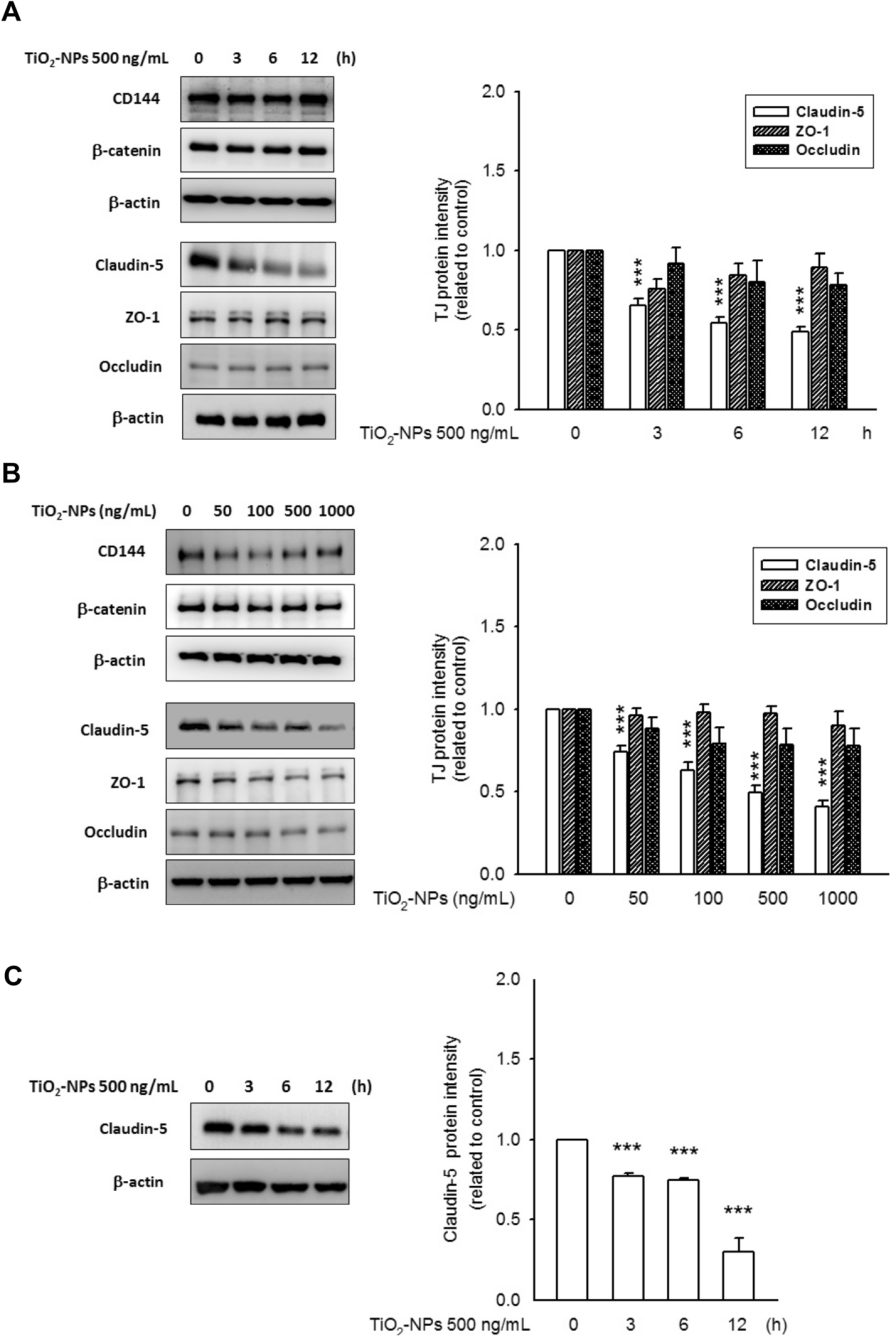
TiO_2_-NP treatment reduced claudin-5 protein level in endothelial cells. Representative images and histograms showed the treatment with TiO_2_-NPs caused an obvious claudin-5 protein degradation in both time-dependent **a** and mass-dependent **b** manner. But, the expression level of ZO-1, occludin, CD144 (VE-cadherin) and β-catenin was remained unaltered. **c** TiO_2_-NP-mediated claudin-5 protein degradation has been affirmed in HREC, suggested the ocular vascular plexus might be the target while TiO_2_-NP exposure. ***p < 0.001, indicates statistically significant difference from the control treatment

bEnd.3 cells, a well-accepted cell model with intact TJ structure [22], were used in this work to understand whether TiO_2_-NP negatively regulates the TJ and AJ constituents. After 3 h of TiO_2_-NP incubation, claudin-5 protein, an influential factor of endothelial TJ assembly, was found to degrade with time. The lowest claudin-5 protein level was observed upon treatment with TiO_2_-NPs for 12 h (Fig. 1a). Further, pronounced caludin-5 protein degradation was also observed in the mass-dependent treatment (50–1000 ng/mL, 12 h) (Fig. 1b). The expression of other TJ proteins (including ZO-1 and occludin) and AJ proteins (CD144 and β-catenin) remained unaltered in both time-course and mass-dependent treatments (Figure S1). Importantly, none of these proteins responded to TiO_2_-MPs (Figure S2), suggesting that the degradation of claudin-5 is unique to TiO_2_-NPs. Additionally, TiO_2_-NP-mediated claudin-5 degradation was also observed in the primary culture of HRECs (Fig. 1c) and human umbilical vein endothelial cells (HUVECs) (data not shown). Upon treatment with 500 ng/mL TiO_2_-NPs for 6 and 12 h, the amount of claudin-5 protein decreased to 0.75 ± 0.01 and 0.30 ± 0.09, respectively, which was dramatically reduced compared to the control level (*p* < 0.001). Due to the disturbance of claudin-5, TiO_2_-NPs might affect the inner BRB structure as well as barrier function. Furthermore, neither TJ nor AJ proteins of RPECs were responsive to TiO_2_-NP treatment (data not shown). Thus, we considered that retinal endothelial cells, rather than RPECs, are vulnerable to TiO_2_-NP exposure.

### TiO2-NP-mediated claudin-5 degradation conferred impairment of TJ barrier function

**Fig. 2:**
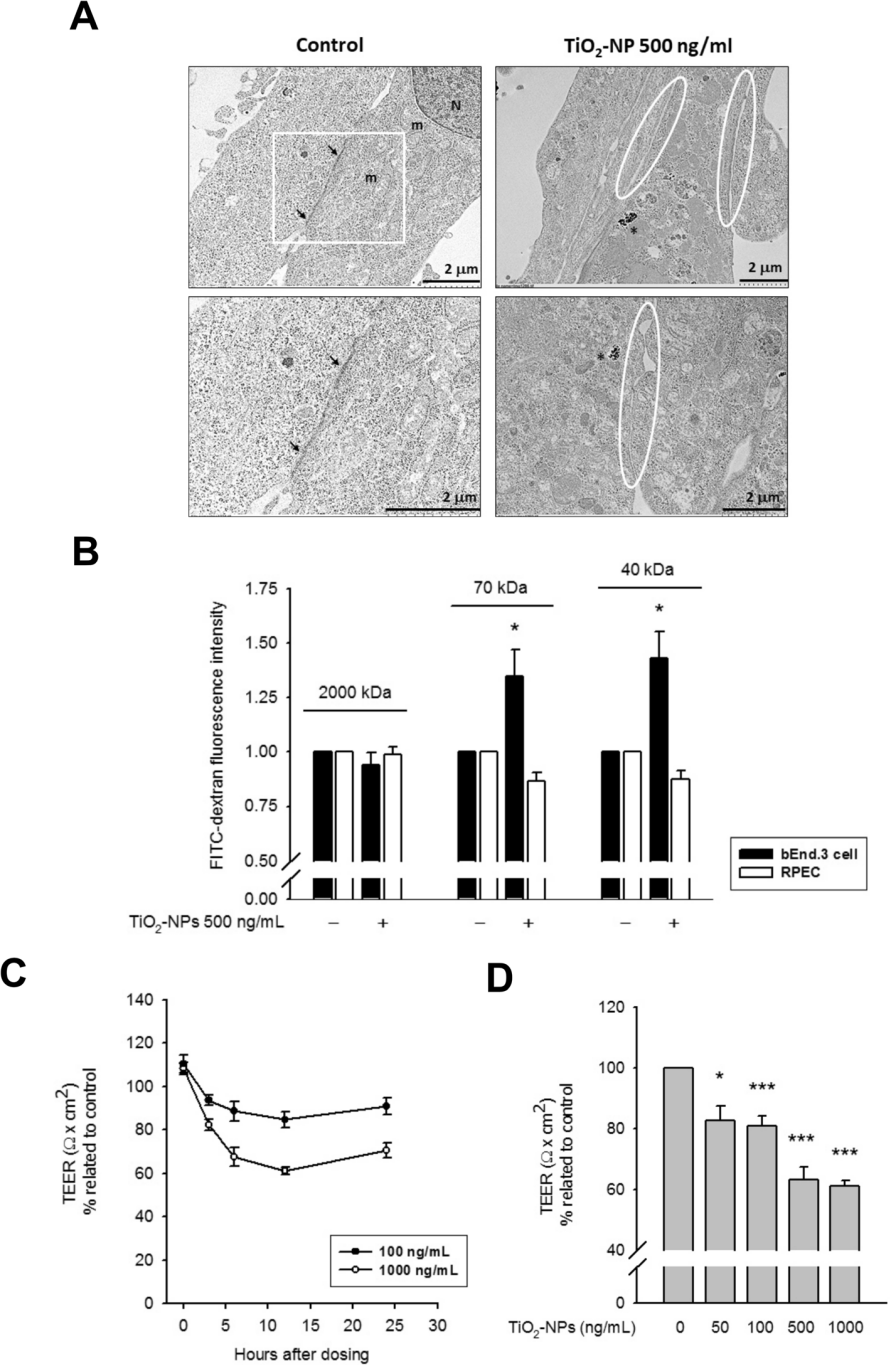
TiO_2_-NP treatment impaired the physiology barrier function of endothelial cells. **a** TEM images showed TJ ultrastructure in the border of adjacent bEnd.3 cells (black arrow). After 12 h incubation with 500 ng/mL TiO_2_-NPs, the appearance of TJ was dramatically reduced, and an enlarged cleft between adjacent cells were observed (indicated by white ellipse). The N and m represents the nucleus and the mitochondria, respectively. Asterisk indicates the engulfed TiO_2_-NPs. **b** Paracellular tracer-flux assays were performed using postconfluent monolayers of bEnd.3 cell and RPEC, as described in Materials and Methods. Treatment with TiO_2_-NPs (500 ng/mL, 12 h) caused an increase in the paracellular permeability of FITC-dextrans (M.W. 40 and 70 kDa); results from 3 to 4 independent experiments are shown. *p < 0.05: significant differences relative to control. **c**/**d** After a treatment of TiO_2_-NPs, TEER was measured (3, 6, 12, 24 h after treatment) between the apical and the basolateral compartment, employing an EVOM ohmmeter. Normalized TEER value (% related to control) was notably reduced after TiO_2_-NP treatment, either in time-dependent manner **c** or in mass-dependent manner **d**. (*N* > 4) *p < 0.05, **p < 0.01, ***p < 0.001, indicates statistically significant difference from the control treatment

Next, the integrity and strength of endothelial TJs were studied. First, as shown in Fig. 2a, the TJ structure at the border of adjacent bEnd.3 cells was observed. Upon TiO_2_-NP treatment (500 ng/mL, 12 h), the TJs almost disappeared, and a gap between adjacent cells was observed, suggesting a loss of the physiological barrier in TiO_2_-NP-treated endothelial cells. An in vitro paracellular permeability assay demonstrated the diffusion of FITC-dextrin (40 and 70 kDa) across the bEnd.3 cell monolayer through the paracellular cleft. However, this cleft was impermeable to 2000 kDa FITC-dextrin. The tolerance of epithelial TJ proteins to TiO_2_-NP treatment was demonstrated by a complete lack of FITC-dextrin diffusion across the RPEC-derived barrier (Fig. 2b). The strength of TJs was also evaluated using TEER, wherein the flux of solutes is restricted in tightly contacted cells, conferring a high TEER value. Following a 3 h TiO_2_-NP incubation, the TEER value of the bEnd.3 cell monolayer was notably reduced with time. The lowest TEER value observed at 12 h was correlated with the claudin-5 expression pattern and was also found to be dose-dependent (Fig. 2c-d). These data confirmed that TiO_2_-NP-mediated claudin-5 degradation may result in the collapse of the physiological barriers composed of endothelial TJs.

### TiO2-NP activates ADAM17 directly, contributing to rapid claudin-5 protein degradation

**Fig. 3:**
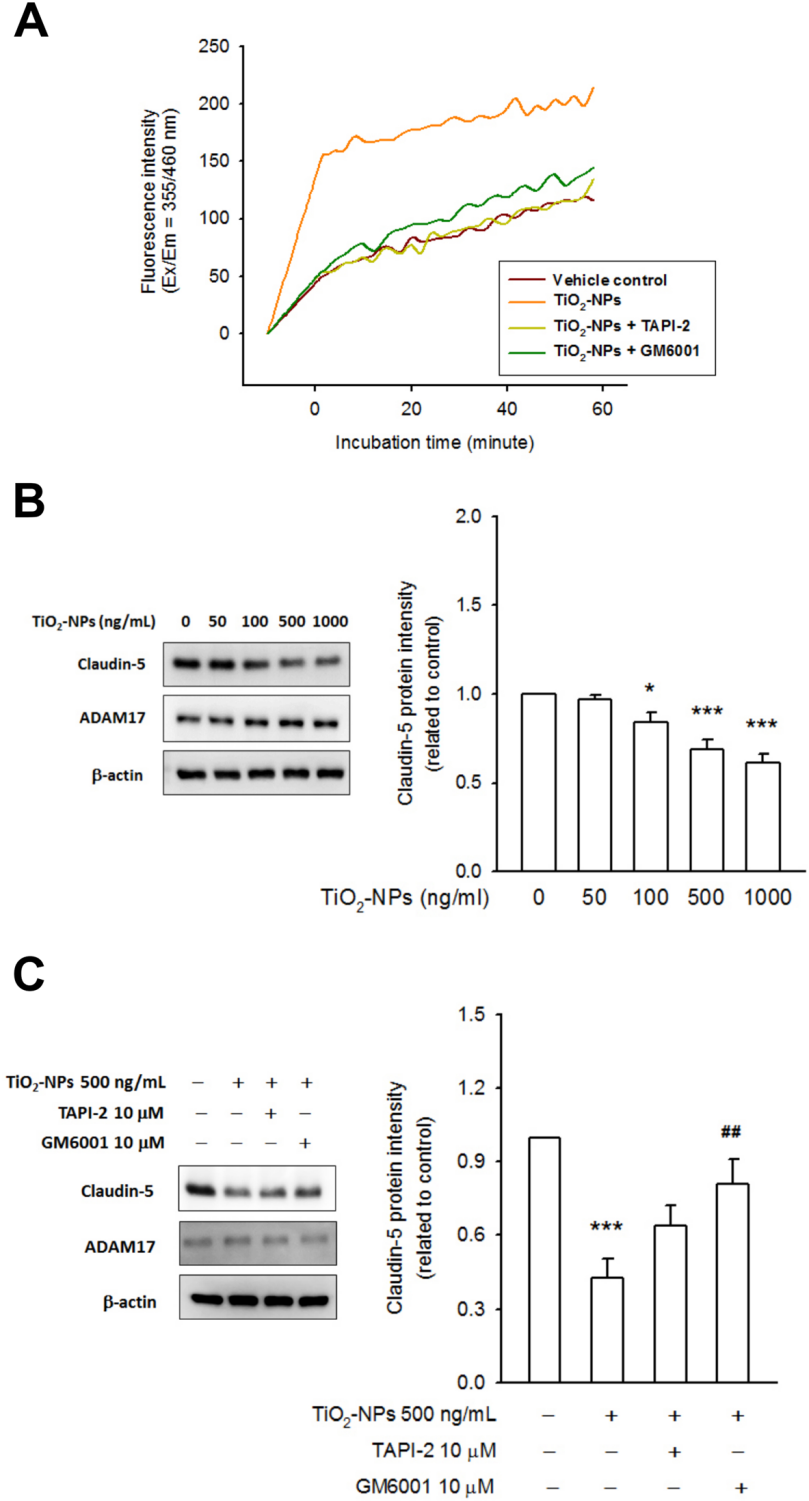
TiO_2_-NP activated ADAM17 directly, contributing to a rapid claudin-5 protein degradation. **a** ADAM17 kinetics assay was performed by fluorometric method in accordance with manufacture’s protocol. The releasing fluorescence from FRET substrate is relative to ADAM17 activity. The basal ADAM17 activity of HREC lysate (vehicle control) was showed in brown curve. In combination with TiO_2_-NP (500 ng/mL), an increasing fluorescence illustrated the induction of ADAM17 activity by TiO_2_-NP (orange curve), whereas the fluorescence signals were prevented by GM6001 (dark green curve) and TAPI-2 (light green curve). **b** HREC lysate was prepared and aliquoted (20 μg protein/vial), then incubated with TiO_2_-NPs (50–1000 ng/mL) at 37 °C for 3 h. Another vial, without TiO_2_-NP treatment, was used as control. Next, the reaction was stopped by the addition of SDS-PAGE sample loading buffer. The amount of claudin-5 was detected by immunoblotting. Data showed an apparent protein degradation of claudin-5, whereas ADAM17 protein level was not changed, whatever the presence or absence of TiO_2_-NP treatment. **c** TiO_2_-NP-mediated claudin-5 degradation could be prevented by GM6001 and TAPI-2. These data provide substantial evidence to support the TiO_2_-NP-mediated claudin-5 degradation, involving the activation of ADAM17. (*p < 0.05, **p < 0.01, ***p < 0.001, indicates statistically significant difference from the control treatment; ## p < 0.01 indicates statistically significant difference from the TiO2_2_-NP-treated group)

We then quantified the transcripts of claudin-5, ZO-1, and occludin. After 4–6 h incubation with TiO_2_-NPs, a conflicting change in their transcripts was found, where ZO-1 and occludin did not change at the protein level (Figure S3). As mentioned above, TiO_2_-mediated claudin-5 degradation was initially observed after 3 h of incubation, suggesting that a rapid proteolysis process might be involved. The kinetics of ADAM17 activity were measured using a fluorometric method. Compared to the ADAM17 positive control, which showed a steady increase in its fluorescence signal over time (data not shown), the kinetics curve of the HREC lysate (or bEnd.3 cell lysate) was flattened. An increase in fluorescence as a function of time was observed upon combination with TiO_2_-NPs; moreover, the presence of GM6001 (a metalloproteinase inhibitor) or TAPI-2 (an ADAM17 inhibitor) strongly abolished the fluorescence kinetics (Fig. 3a and Figure S4A). These data indicated that TiO_2_-NPs could activate ADAM17 directly. To determine the effect of TiO_2_-NP-induced ADAM17 activation on claudin-5 degradation, the HREC lysate was added to vials along with TiO_2_-NPs. Following a 3 h incubation at 37 °C, the amount of claudin-5 was determined by immunoblotting. We found pronounced degradation of claudin-5 in a TiO_2_-NP-dependent manner compared to the vehicle control, but did not affect the amount of ADAM17 (Fig. 3b). Moreover, claudin-5 degradation was prevented by adding GM6001 and TAPI-2 (Fig. 3c). Similar results were obtained for the bEnd.3 cell lysate (Figure S4). These data indicated that TiO_2_-NPs could activate ADAM17 immediately, contributing to the rapid degradation of claudin-5 protein.

### TiO2-NP-mediated ADAM17 activation improved endothelial cell migration

**Fig. 4:**
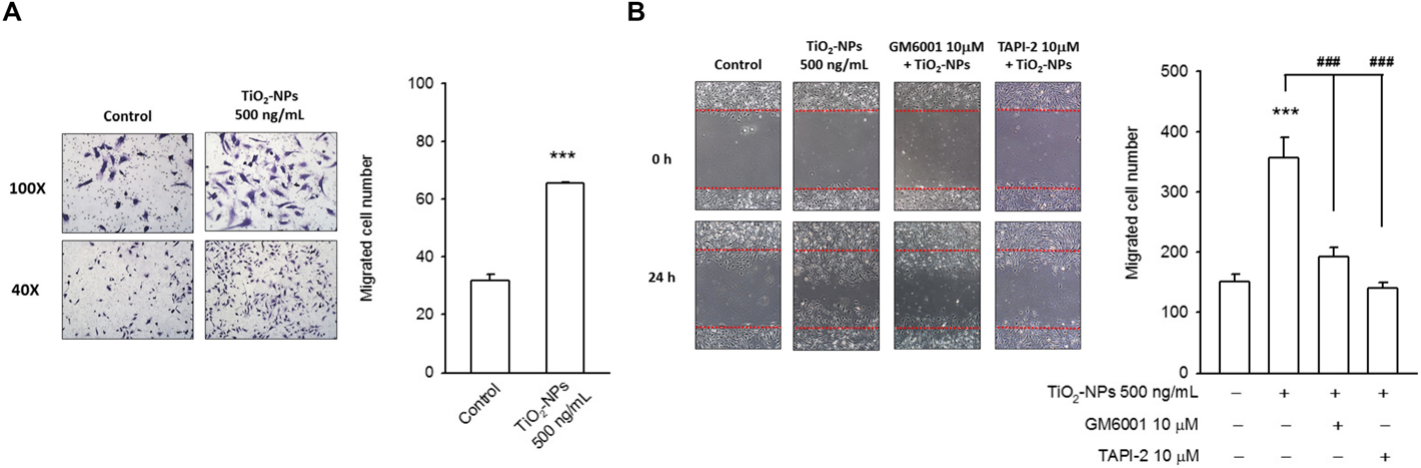
TiO_2_-NP-mediated ADAM17 activation improved endothelial cell migration. Both the representative images and quantified data demonstrated TiO_2_-NP improved endothelial cell migration. This phenomenon could be prevented in the presence of GM6001 or TAPI-2. **a** Transwell assay. **b** Scratch assay. (*p < 0.05, **p < 0.01, ***p < 0.001, indicates statistically significant difference from the control treatment; ### p < 0.001 indicates statistically significant difference from the TiO_2_-NP-treated group)

Claudin-5 has also been identified as a factor required for cell-cell contact and negatively regulates cell migration [23]**.** After 12 h of TiO_2_-NP incubation, endothelial cells were found capable of migrating both in the Transwell assay (Fig. 4a) and wound healing assay (Fig. 4b). TiO_2_-NP-mediated cell migration was reversed, whereas the presence of GM6001 or TAPI-2 (Fig. 4b), suggesting the involvement of ADAM17 in TiO_2_-NP-mediated endothelial cell migration.

### Claudin-5 turnover was postponed in ADAM17-knockdown bEnd.3 cells

**Fig. 5:**
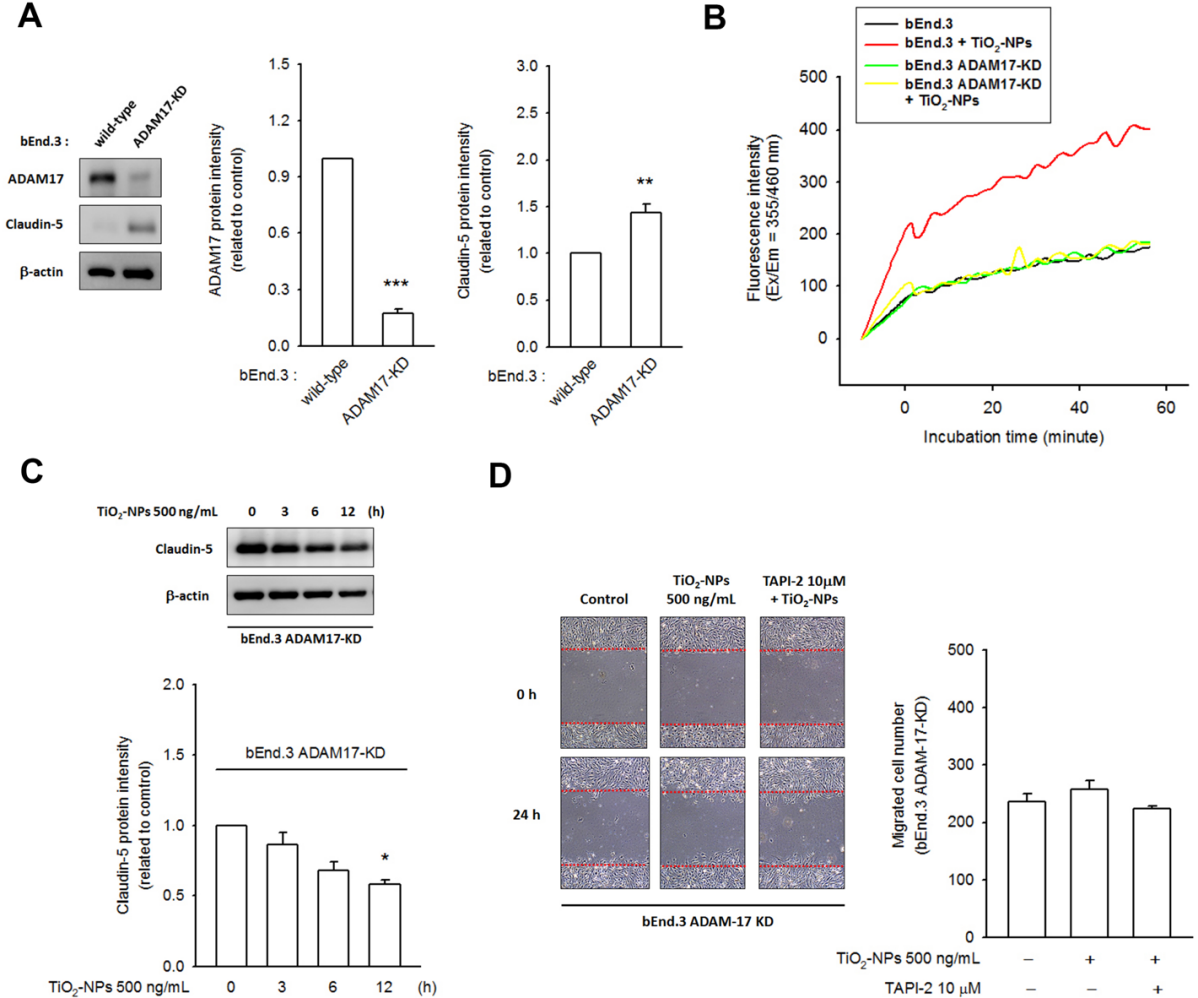
Claudin-5 turnover was postponed in ADAM17-knockdown bEnd.3 cells. **a** bEnd.3 stable clones with ADAM17-knockdown were built and the claudin-5 expression level was greatly elevated in these clones. **b** In bEnd.3 ADAM17-KD clones, the enzymatic kinetics of ADAM17 was not induced in responded to TiO_2_-NP treatment. **c** TiO_2_-NP-mediated claudin-5 degradation was strongly reduced in bEnd.3 ADAM17-KD cells. **d** TiO_2_-NP-mediated cell migration was also alleviated in bEnd.3 ADAM17-KD cells. *p < 0.05, **p < 0.01, ***p < 0.001, indicates statistically significant difference from the control treatment

Next, the role of ADAM17 in regulating claudin-5 turnover was evaluated using ADAM17-knockdown bEnd.3 cells. As shown in Fig. 5a, successful knockdown of ADAM17 coincided with claudin-5 upregulation. As predicted, the ADAM17 activity of bEnd.3 ADAM17-KD did not respond to TiO_2_-NP treatment (Fig. 5b). Subsequently, TiO_2_-NP-mediated claudin-5 degradation (Fig. 5c) and TiO_2_-NP-mediated cell migration (Fig. 5d) was notably prevented by ADAM17 knockdown. Thus, we considered ADAM17 as a crucial factor required for maintaining the endothelial physiology barrier function by regulating claudin-5 turnover.

### Intravitreal exposure of TiO2-NPs disrupted the BRB and alleviated ocular blood flow in vivo

**Fig. 6:**
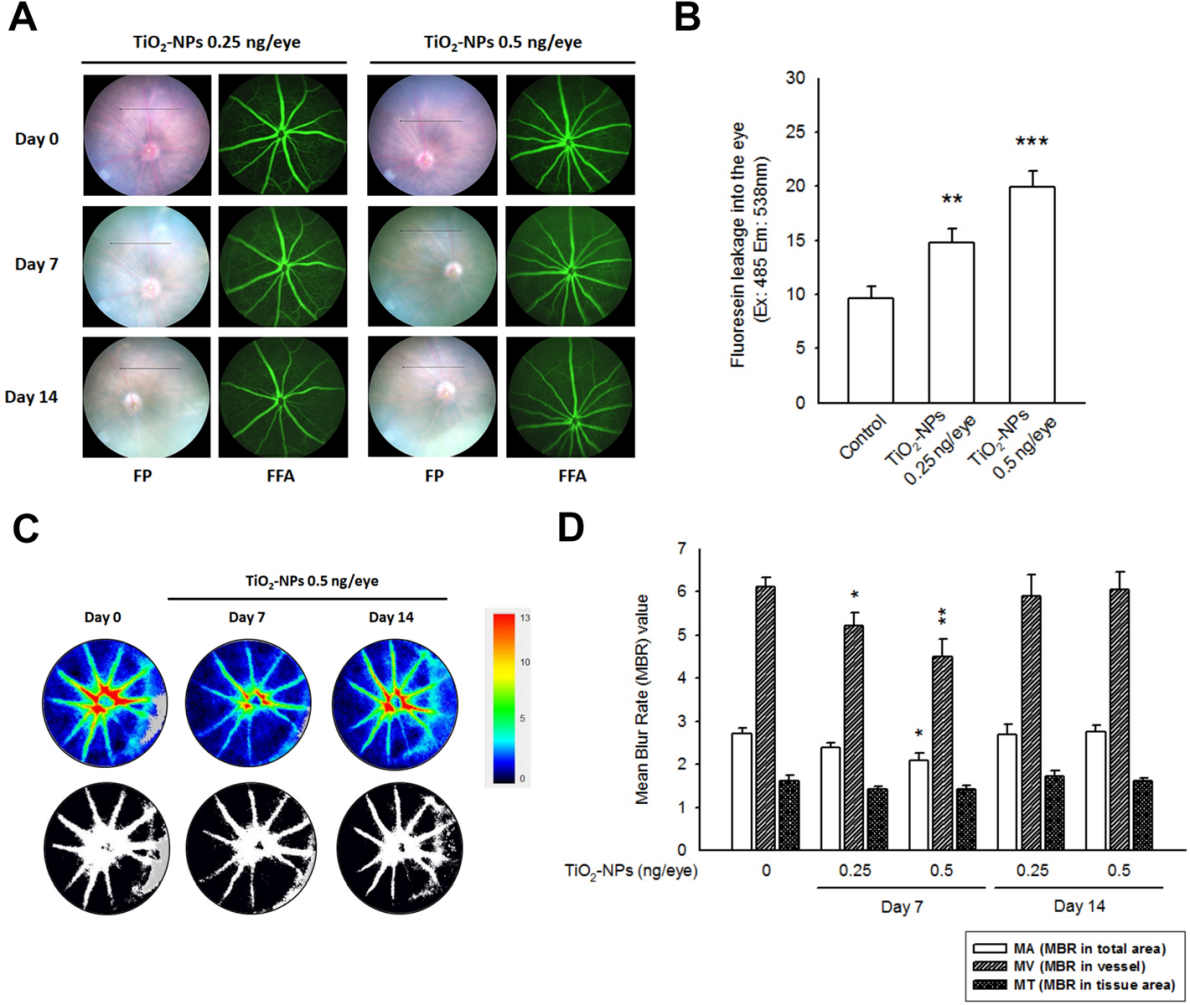
Intravitreal exposure of TiO_2_-NPs broken BRB and alleviated ocular blood flow in vivo. **a** In the group receiving intravitreal TiO_2_-NP, the treated right eye revealed leaking of fluorescein from the vessel to vitreous body, resulting an obscure view of the fundus. **b** Next day, the eyeballs were isolated and homogenized. An accumulation of fluorescein was found in the extracts, suggested the intravitreal TiO_2_-NP might cause the leakage of inner BBB. **p < 0.01, ***p < 0.001, indicates statistically significant difference from the control eyeballs (left eye). **c**-**d** Measurement and analyzing the MBR values in the ONH circulation in C57BL/6 mice before (day 0) and after (day 7 and 14) a single-dose TiO_2_-NP treatment. **c** Representative color-coded maps of the MBR (upper panel). By using definitive threshold, the ONH vessel region (shown in white), and the ONH tissue region (shown in black) were automatically defined and showed in gray-scale maps (lower panel). **d** The calculated MBR values of the total retinal regions (MA), the vessel regions (MV), and the tissue region (MT) that shown as mean ± S.E.M., was presented in histograms. MBR values of MA and MV were significantly reduced in TiO_2_-NP-treated groups at day 7, whereas the MBR values were returned to basal level on day 14. *p < 0.05, **p < 0.01, ***p < 0.001, indicates statistically significant difference from the control (day 0)

To observe the changes in ocular parameters in vivo, C57BL/6 mice were intravitreally administered a single dose of TiO_2_-NPs (0.25 and 0.5 ng/eye; for the vitreous volume of mice [24], the dosing level was equivalent to 50 and 100 ng/mL), and subjected to a retinal function observational battery (Figure S5). The mean IOP of TiO_2_-NP-treated eyes showed no significant difference on day 7, whereas a decrease in mean IOP was noted in the 0.5 ng/eye treated group on day 14 (Table S3). Considering the loss of claudin-5 protein and an increase in paracellular permeability observed in vitro, it is necessary to study the changes in BRB in vivo. FFA images before dosing (day 0) showed uniform filling of the perfused fluorescein in retinal vessels at the ONH, where the length and diameter of the vessels were apparent. However, after TiO_2_-NP treatment, fluorescein leakage was observed, which consequently dimmed the view of the fundus (Fig. 6a). The eyeballs were then isolated and homogenized on the next day, followed by fluorescein intensity quantification. Generally speaking, the injected sodium fluorescein was completely eliminated after 24 h, whereas the leaking fluorescein remaining in the vitreous body was excluded slowly [25]. Quantitative data showed a dose-dependent accumulation of fluorescein in TiO_2_-NP-treated eyeballs (right eye), compared to the vehicle-treated eyeballs (left eye), supporting that leakage of the inner BRB was induced by TiO_2_-NPs (Fig. 6b).

The ocular blood flow of the ONH was measured consecutively using the LSFG method. Figure 6c presents color-coded maps of the MBR, showing a reduction in ocular blood flow at the ONH on day 7 post dosing (Fig. 6d). Three MBR values (MA, MV, and MT) expressed in different regions were significantly reduced in the TiO_2_-NP-treated groups at day 7, with a dose-dependent relationship. MA represents the average MBR over the entire ONH, whereas the MV and MT represent the average of the vessel region and tissue region, respectively. However, the MBR values returned to normal levels at day 14. Similar results were obtained with the MBR measurements of the choroid (Figure S6).

### Intravitreal exposure to TiO2-NPs impaired the retinal electrophysiology

**Fig. 7:**
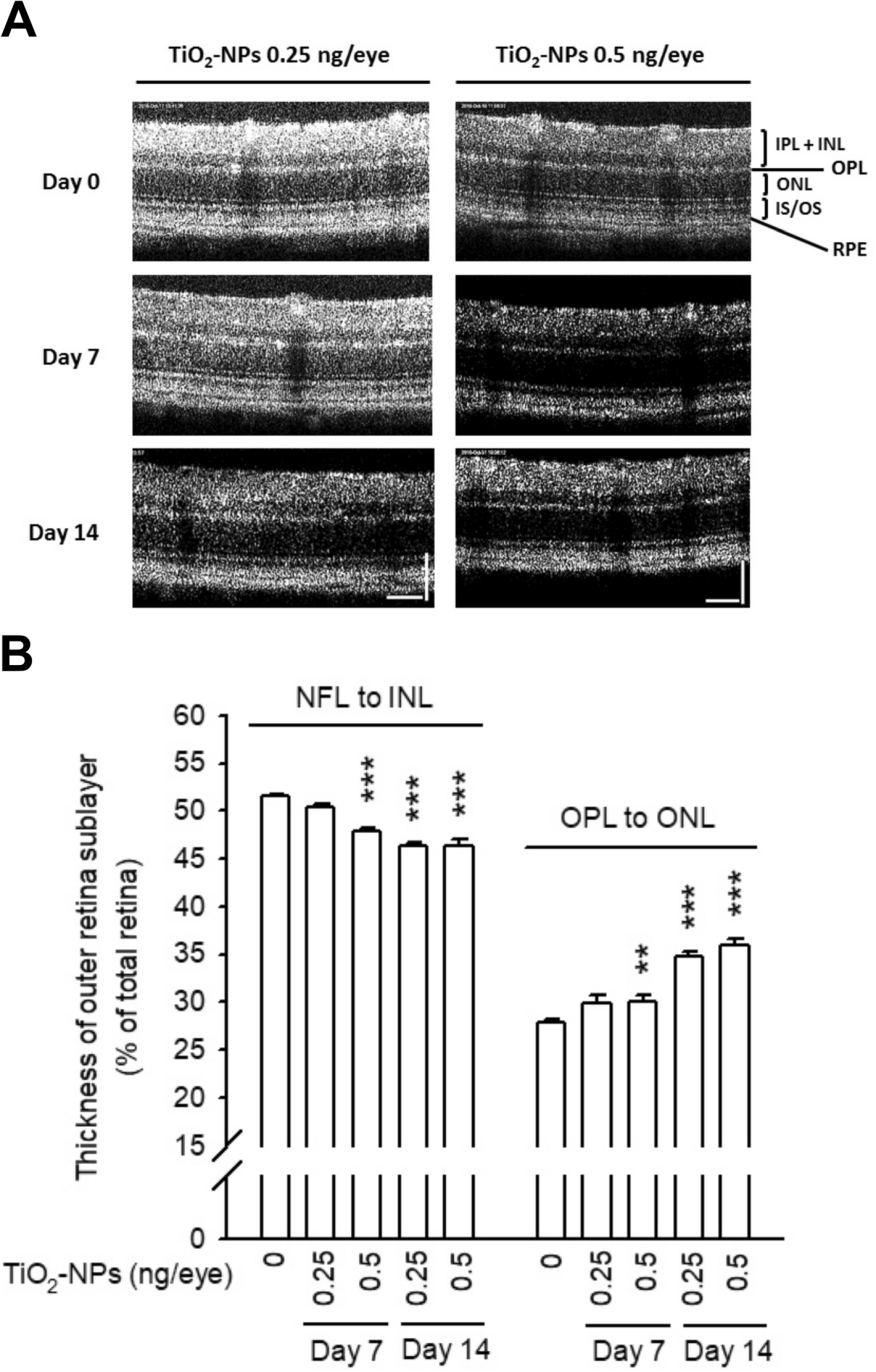
Intravitreal exposure of TiO_2_-NPs changed the thickness of retinal sublayers. **a** SD-OCT scan images were taken from the mice, employing the Micron III retinal imaging microscope. No significant damages were identified in OCT images. **b** Following the setting of InSight software, the thickness of three retinal sublayers was automatically identified and computed, and expressed as % of total retina thickness. The thickness of inner sublayer, including the nerve fibers layer (NFL), the ganglions, the inner plexiform layer (IPL) and the inner nuclear layer (INL), was reduced at day 7 (0.5 ng/eye) and day 14 (0.25 and 0.5 ng/eye). But, an increased thickness of middle sublayer, comprises the outer plexiform layer (OPL) and the outer nuclear layer (ONL), has been computed at day 7 (0.5 ng/eye) and day 14 (0.25 and 0.5 ng/eye). **p < 0.01, ***p < 0.001, indicates statistically significant difference from the control (day 0)

**Fig. 8:**
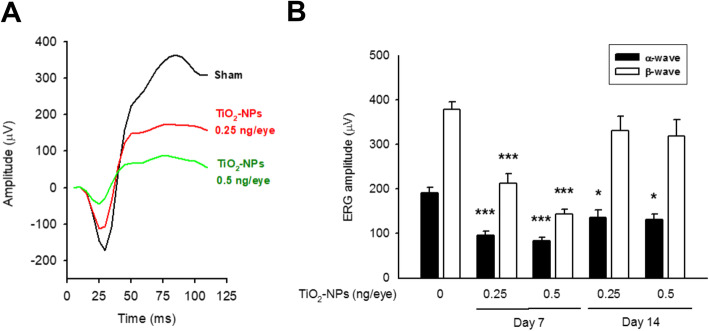
Intravitreal exposure of TiO_2_-NPs impaired the electrophysiology of retina. **a** ERG recordings were performed in dark room as described in Materials and Methods. Representative ERG α- and β-wave showed damages in their amplitude after TiO_2_-NP treatments (green and red lines), as compared to sham (black line). **b** Quantified data showed a great reduced amplitude of at day 7 (both α- and β-wave) and day 14 (α-wave); whereas β-wave amplitude seemed recovery at day 14. *p < 0.05, ***p < 0.001, indicates statistically significant difference from the control (day 0)

Retinal structure was determined by SD-OCT, and the thickness of the total retina as well as retinal sublayers, were computed using software. Neither retinal structure nor the thickness of total retina was observed changed by OCT scan images before (day 0) and after TiO_2_-NP treatment (Fig. 7a; Figure S7A). However, an inappreciable atrophy was identified in the inner retinal sublayer (from the NFL to the INL) at day 7 (47.87 ± 0.42% of total retina thickness, 0.5 ng/eye treated group) compared to that on day 0 (51.51 ± 0.26%). The middle retina sublayer (the OPL and ONL) was showed an increased thickness on day 7 (30.13 ± 0.59% of total retina thickness, 0.5 ng/eye treated group) compared to that on day 0 (27.88 ± 0.32%). These changes were sustained at day 14 (Fig. 7b). An inappreciable reduction in the outer sublayer, comprises the inner and outer segments (IS/OS) of photoreceptors, was also computed at day 14 (0.5 ng/eye) (Figure S7B). Finally, the ERG performance was recorded to understand the visual transduction driven by retinal neurons. The mean amplitudes of α- and β-waves were dramatically alleviated in TiO_2_-NP-treated groups at day 7, and the damage was correlated to the administered dose. The β-wave amplitude recovered at day 14, whereas the α-wave amplitude was partially ameliorated (Fig. 8a-b). No differences were found in implicit time (data not shown). These data strongly indicate that TiO_2_-NPs impaired the ERG amplitude in vivo.

## Discussion

TiO_2_-NPs, one of the most manufactured nanomaterials worldwide, is primarily used in the cosmetic industry. Approximately 20–40% of the initial amount of TiO_2_-NP was found to be released from sunscreen-applied skin after 120 min of stirring [26]. In addition, large amounts of TiO_2_-NPs are frequently present in swimming water (21–60 μg/L) and in surface water (2–700 ng/L) [27, 28]. This makes eye-contact with TiO_2_-NPs inevitable. Owing to the presence of numerous ocular barriers composed of peculiar constituents, it is difficult to deliver drug molecules into the eyeballs, especially in the posterior region. The cornea-conjunctiva barrier has multiple layers of tightly compacted epithelial cells and effectively limits the ocular penetration of molecules from topical administration. Less than 5% of the total administered molecules can reach the aqueous humor [29]. Currently, drug delivery to ocular tissues using NPs is attractive because they not only improve bioavailability but also prolong residence time [30, 31]. For example, after topical instillation, NPs comprised of different materials (40–220 nm) could be traced in vitreous fluid and retina of mice within a short time, suggesting the potential to pass the cornea-conjunctiva barrier [32–34]. Once reaching the vitreous body, the diffusivity of particles is restricted by the nature of vitreous gel, which is composed of a collagen fibril network and hydrated glycosaminoglycans random coils with negative charge [14]. Only particles with a diameter < 510 nm and possessing neural or negative surface charge could diffuse readily into the vitreous body [14, 35]. A number of studies have reported that with appropriate size and surface charge, intraocular NPs could gradually spread throughout the retinal layers, and reside there for up to 90 days post-dosing [36–39]. Our preliminary data demonstrated the successful penetration of TiO_2_-NP from the corneal surface to the posterior region of the eye after topical administration; however, the efficiency varied widely among individuals (data not shown). Repetitive exposure increases the level of TiO_2_-NP, and when the accumulation reaches a hazardous level, intraocular damage occurs. To understand the correlation between the observable retina injuries and dosing level, the ITV injection was used in this study. Following a single-dose ITV injection of TiO_2_-NPs, the light-adapted electrical activity generated by retinal cells was impaired in vivo, wherein the α-wave amplitude was reduced significantly until day 14. This change reflects the damage in the hyperpolarization response of the photoreceptors; subsequently, the bipolar cells and Muller cells were reduced in the depolarization, as indicated by the recording of the minimalized amplitude of β-waves. Additionally, either fluorescein accumulation in the vitreous body, or decreased blood flow in the ONH, as observed in TiO_2_-NP-treated eyes, indicated the leakage of retinal microvessels.

To maintain visual function, it is essential to preserve homeostasis of the retina and sub-retinal space by restricting the flow of solutes, nutrients, wastes, proteins, and water flux in and out of the retina. Two restrictive physiological barriers, the inner BRB (composed of retinal endothelial cells) and outer BRB (comprising RPECs), are particularly involved in this process. The cytotoxicity of TiO_2_-NP varied among cell types, and in part, was attributed to the crystallographic form of TiO_2_-NPs. It was shown that the rutile form had a stronger cytotoxic effect than the others [40]. In agreement with previous in vitro studies involving constituent cells of the retina and endothelial cells, TiO_2_-NP-mediated cytotoxicity occurred at concentrations > 5 μg/mL [8–10, 13, 40]; and in our study, we observed that neither RPEC nor bEnd.3 cell death occurred by TiO_2_-NPs. This indicated that the impairment of retinal function might not be attributed to cytotoxicity. In this study, we found that TiO_2_-NP treatment reduced the claudin-5 protein level of endothelial cells, conferring an increase in paracellular permeability, a decline in TEER value, and an improved migration ability in vitro, coincident with retinal microvessel leakage in vivo. However, the TJ components in RPECs did not respond to TiO_2_-NP treatment. These observations indicate that the endothelial cells of the retina are targeted by TiO_2_-NPs.

Next, several studies have suggested that TiO_2_-NPs are capable of producing ROS, and consequently, inflammatory responses relative to ROS are evoked [10, 40]. A number of pro-inflammatory cytokines have been known to affect the strength and structure of TJs as well as the TJ-based physiology barrier function [17, 18]. More importantly, claudin-5 protein degradation is rapidly responsive to TiO_2_-NP treatment, whereas a conflicting result was obtained for claudin-5 mRNA, indicating the involvement of a proteinase. After comprehensively testing a series of proteinase inhibitors, we found that TiO_2_-NP-mediated claudin-5 degradation could be prevented by chemical inhibitors of ADAM17 (GM6001 and TAPI-2). ADAM-17, also called the TNF-α-converting enzyme (TACE), is frequently expressed in endothelial cells where it serves as the chief enzyme to shed the TNF-α precursor to its mature form [41]. Additionally, ADAM17 is responsible for the proteolytic cleavage of a number of membrane-tethered protein substrates, including chemokines and their receptors [42, 43], adhesion molecules [44, 45], and TJ proteins [46–48], thus regulating their subsequent activities and functions. For example, inflammatory cytokines increased ADAM17 activity, contributing to junctional adhesion molecule A (JAM-A) cleavage in the aorta. Downregulation of surface JAM-A destabilized the TJs, whereas shedding of JAM-A repressed the migration of endothelial cells and leukocyte infiltration in vitro [46]. After hypoxia stimulation, the diminished localization of claudin-5 from the endothelial cell membrane and the consequent loss of barrier properties were completely reversed by the inhibition of ADAM17 and ADAM12 activities [48]. ADAM17 defects or activity loss preserved the TJ protein (JAM-A and claudin-5) expression level in vitro and protected the integrity of the intimal barrier in vivo [49]. In our study, we demonstrated for the first time that TiO_2_-NPs could evoke ADAM17 activation immediately. TiO_2_-NP-induced ADAM17 activation readily caused claudin-5 degradation in vials in a mass-dependent manner. In ADAM17-knockdown bEnd.3 cells, the basal level of claudin-5 was boosted; moreover, the amount of claudin-5 did not respond to TiO_2_-NP treatment, as compared to that in wild-type cells. These data indicated that cell-surface claudin-5 undergoes constitutive cleavage, and that TiO_2_-NP treatment dramatically accelerates this turnover process, which is mediated predominantly by ADAM17 activity.

In biological fluids, NPs show high affinity for cell membranes and proteins. TiO_2_-NPs have been demonstrated to adsorb biomolecules (e.g., serum albumin, immunoglobulins, and surfactant-associated proteins) on their surface, forming a protein corona [50, 51]. The interaction between TiO_2_-NPs and lipids/proteins in the cell membrane was proven to build up a lipid covering (the membrane corona around the NP), leading to disruption of the cell membrane or facilitating the cellular internalization of NPs [52]. Depending on these properties, it is reasonable that TiO_2_-NPs are capable of interacting with lipid bilayers and membrane-anchored ADAM17, eventually corresponding to ADAM17 activation. The expression pattern of ADAM17 is predominantly present in the corneal epithelium and retinal vascular plexus. ADAM17 is crucial for processing Notch1 signaling, which influences eye morphogenesis. Mice expressing lower amounts of ADAM17 displayed multiple eye malformations, especially a narrow retinal vasculature [53]. Overall, the expression and activity of ADAM17 is known to contribute to a variety of vascular-related diseases. Thus, ADAM17 may serve as a potential therapeutic target for retinopathy.

## Conclusion

Following ITV injection, we surmised that the TiO_2_-NPs could readily diffuse from the injected site to the central retinal artery branches inside the vitreous compartment and the whole retina. We found, for the first time, that TiO_2_-NPs could activate ADAM17 at ng/ml levels, followed by ADAM17-mediated claudin-5 degradation in a few hours. In light of claudin-5 degradation, we confirmed the collapse of endothelial barriers and enhancement of endothelial cell migration in vitro. The human retina is furnished with a complex vascular plexus, where ADAM17 expression is enriched, suggesting that the retina might be a vulnerable target of TiO_2_-NPs. By ITV administration of TiO_2_-NPs (0.25 ng/eye of mice), injuries to the retinal structure and function were identified in vivo, determining the hazardous level of TiO_2_-NP in ocular tissues. Finally, although TiO_2_-NPs are biologically active in the retina, it cannot be immediately extrapolated to topically applied TiO_2_-NP-based sunscreens, unless a comprehensive understanding of the penetration efficiency of TiO_2_-NPs through topical application is achieved.

## Materials and methods

### TiO2 particles and chemicals

TiO_2_-NPs were acquired from the First Cosmetics Manufacture Co., Ltd. (Taoyuan, Taiwan), whereas TiO_2_-MPs were purchased from Sigma-Aldrich (St. Louis, MO). A TiO_2_ stock suspension of 10 mg/mL was prepared in 30% glycerol, and the working stocks (from 5 to 500 μg/mL) were prepared by serially diluting the stock suspension in serum-free culture media (for in vitro treatment) or in phosphate-buffered saline (PBS, for in vivo treatment). A routine sonication protocol using a probe sonicator (125 W, 20 kHz, amplitude 30%, 8 s/pulse, for 2–3 pulses; QSonica, Newtown, CT), was performed prior to DLS and ζ-potential measurement as well as experimental treatment.

### Cell culture

The bEnd.3 cell line (an immortalized mouse cerebral vascular endothelial cell) and ARPE-19 cell line (human adult RPEC) were obtained from the Bioresource Collection and Research Center (BCRC, Hsinchu, Taiwan). Both cell lines were cultured in Dulbecco’s modified Eagle medium (DMEM) supplemented with 10% FBS, 2 mM L-glutamine, and 1% antibiotic mixture. Primary cultures of human retinal microvascular endothelial cells (HRECs) were purchased from CellBiologics (Chicago, IL). Cryopreserved cells were thawed and propagated on gelatin-coated dishes with complete growth medium from Cell Biologics. Subculture was performed at 80–90% confluence and the medium was refreshed every 2 days.

### Cell viability determination (MTT assay)

Stock cells were seeded in 48-well plates at a density of 3 × 10^4^ cells per well. The next day, the medium was replaced with medium containing TiO_2_-NPs at different concentrations and incubated for 24 h. Metabolically active cells that successfully reduced the yellow tetrazolium salt to insoluble formazan were quantified by colorimetric absorbance at 570 nm.

### Immunoblotting

To understand changes at the protein level, whole cell lysates prepared in radioimmunoprecipitation assay (RIPA) buffer were subjected to SDS-PAGE and western blotting, as described elsewhere. The following antibodies were used in this study: anti-claudin-5, anti-ADAM17 (Abcam, Cambridge, UK), anti-CD144, anti-β-catenin (Genetex, Irvine, CA), anti-occludin (Proteintech, Chicago, IL), anti-ZO-1 (Invitrogen, Waltham, MA), and anti-β-actin (Sigma-Aldrich, Chicago, IL). Densitometry of protein bands was performed using the Gel-Pro Analyzer software (Media Cybernetics, Rockville, MD, USA). β-actin was used as the loading control.

### Transmission electron microscopy (TEM)

Samples for TEM were processed in accordance with the standard protocol of the TMU imaging core facility. TEM images were obtained using a Hitachi HT-7700.

### Real-time quantitative polymerase chain reaction (qPCR) analysis

Genes of interest were quantified at the mRNA level by real-time qPCR. The protocol and primer sequences for PCR are listed in the Supplementary Information.

### Evaluation of TJ barrier function

The physiological barrier function of TJ was evaluated in vitro by paracellular permeability to FITC-dextran and by transendothelial electrical resistance (TEER). Both assays required a post-confluent cell monolayer grown on the Transwell upper chamber (0.4 μm pore size). For the paracellular permeability measurement, 200 μg/mL FITC-dextran (40, 70, and 2000 kDa) was added into the upper chamber. The diffusion of FITC-dextran to the outer chamber was then measured after 30 min at 485/535 nm (Ex/Em) using a multi-mode plate reader (Fluoroskan Ascent FL, Thermo Fisher Scientific, Waltham, MA). TEER values were acquired using an EVOM™ Epithelial Voltohmmeter (World Precision Instruments; Sarasota, FL, USA) as described previously [54]. Normalized TEER values were corrected for background and expressed as Ω·cm2.

### ADAM17 (TACE) enzymatic activity

ADAM17 activity was assayed by the release of the quenched fluorescence group from a specific FRET substrate (BioVision, Milpitas, CA). The whole cell lysate was aliquoted (20 μg/well) in commercial assay buffer. After a short incubation at 37 °C, the FRET substrate was added to each well. The fluorescence generated by substrate hydrolyzation was then recorded kinetically for 60 min at 318/449 nm (Ex/Em).

### Cell migration

Both the Transwell and scratch assays were performed to evaluate the migration capacity of TiO_2_-NP-treated cells. In the Transwell assay, cells (5 × 10^3^ cells/well) were placed in the upper chamber (8-μm pore size), followed by incubation with TiO_2_-NPs for 12 h. The migrated cells on the opposite side of the filter were fixed, stained, and counted. For the wound healing assay, using a silicone insert, a scratch was made on the cell monolayer. After TiO_2_-NP treatment, cells were monitored and counted as they moved into the gap.

### Animal husbandry and treatment

C57BL/6 mice (6–8 weeks old) obtained from an AAALAC certified supplier were grouped randomly (6–10 mice/group) and housed at Taipei Medical University Animal Center. The animal handling protocol was approved by the facility IACUC (LAC-2014-0242), and the experimental flowchart is shown in Figure S1. On day 0, the retinal function observational battery, including intraocular pressure (IOP), fundus photography (FP), fundus fluorescein angiography (FFA), laser speckle flowgraphy (LSFG), optical coherence tomography (OCT), and electroretinogram (ERG), was performed in all animals, and the results represented the control status. Next, the mice in the testing groups received a single-dose, ITV injection of TiO_2_-NPs (0.25 and 0.5 ng/eye) in their right eye, whereas a vehicle injection was performed in the sham group. The injected volume was 1 μL per eye. At day 7 and 14 post dosing, retinal function was re-evaluated as described earlier.

### Fundus photography, fundus fluorescein angiography, and spectral domain-optical coherence tomography

After anesthesia (ketamine 80 mg/kg and xylazine 20 mg/kg), the right eye of the mouse was dilated with 0.125% atropine and treated with a drop of 2% Methocel® (OmniVision, Switzerland). Using an ophthalmoscope, the ocular fundus was focused and positioned circumferentially around the optic disc; FP and FFA images were then acquired using the Phoenix Micron III retinal imaging system (Tempe, AZ). An intraperitoneal injection of sodium fluorescein (10 mg/kg) is essential prior to FFA administration. Following the manufacturer’s instructions, SD-OCT images were scanned vertically or horizontally at the indicated positions. The thickness of the three retinal sublayers was automatically identified and computed using the InSight software. The inner sublayer comprises the nerve fiber layer, ganglion layer, inner plexiform layer (IPL), and inner nuclear layer (INL). The middle sublayer comprises the outer plexiform layer (OPL) and outer nuclear layer (ONL), whereas the outer sublayer comprises the inner and outer segments (IS/OS) of photoreceptors [55].

### Retinal blood flow measurement by laser speckle flowgraphy (LSFG)

The mean blur rate (MBR), a relative index of blood velocity, was measured using laser speckle flowgraphy (LSFG). Briefly, anesthetized mice were placed horizontally. Their right eyes were dilated and prepared as described earlier. After focusing, the MBR images were acquired continuously at a rate of 30 frames per second over a 4-s period. Several MBR values were automatically calculated using the equipped software (version 3.1.14.0; Softcare Co., Ltd., Japan).

### Electroretinogram (ERG)

Anesthetized mice with dilated pupils were placed in a dark room. A reference electrode and a ground electrode were placed at the center of the scalp and proximal tail skin, respectively. The third electrode was placed in contact with the corneal surface. After the light stimulus, both α-waves and β-waves were recorded and amplified using the MP36 4-channel data acquisition system (BIOPAC Systems, UK) [55]. The amplitude and implicit time of the α- and β-waves were then analyzed, and the overall average was expressed.

#### Statistical analysis

All data were expressed as mean ± standard error of the mean (S.E.M). Statistical differences among groups were tested using one-way analysis of variance (ANOVA) or Student’s t-test. *p < 0.05 and **p < 0.01 or ***p < 0.001 represents statistical significance.

## Supplementary Information

**Additional file 1: Table S1.** Characteristics of TiO_2_ particles. **Table S2.** Effects of TiO_2_ particles on cell viability (MTT assay). **Table S3.** Observations on mice intraocular pressure (IOP) of intravitreal treatment with TiO_2_-NP.

**Additional file 2: Figure S1.** TiO_2_-NPs did not affect the expression of AJ proteins. **Figure S2.** TiO_2_-MPs did not affect the expression of TJ/AJ proteins. **Figure S3.** Changes in mRNA level of claudin-5, ZO-1 and occludin in TiO2_2_-NP-treated bEnd.3 cells. **Figure S4.** TiO_2_-NP activated ADAM17 directly, contributing to rapid claudin-5 protein degradation. **Figure S5.** Scheme of animal treatment. **Figure S6.** Reduction of choroid blood flow was evidenced in TiO_2_-NP-treated mice. **Figure S7.** Intravitreal exposure of TiO_2_-NPs changed the thickness of retinal sublayers.

## Data Availability

The datasets used and/or analyzed during the current study are available from the corresponding author upon reasonable request.

## Abbreviations

TiO_2_-NPs: Titanium dioxide nanoparticles
MPs: Microparticles
BRB: Blood-retinal barrier
RPEC: Retinal pigment epithelial cell
ONH: Optic nerve head
LSFG: Laser speckle flowgraphy
MBR: Mean blur rate
UV: Ultraviolet
TNF-α: Tumor necrosis factor-α
TACE: TNF-α converting enzyme
ADAM17: A disintegrin and metalloproteinase 17
ZO-1: Zonula occludens-1
TEER: Transendothelial electrical resistance
ITV: Intravitreal

## References

[1] Sharma S, Sharma RK, Gaur K, Cátala Torres JF, Loza-Rosas SA, Torres A, et al. Fueling a hot debate on the application of TiO2 nanoparticles in sunscreen. Materials (Basel). 2019:12. 10.3390/ma12142317 PMC6678326 31330764

[2] Mohajerani A, Burnett L, Smith JV, Kurmus H, Milas J, Arulrajah A, et al. Nanoparticles in construction materials and other applications, and implications of nanoparticle use. Materials (Basel). 2019:12. 10.3390/ma12193052 PMC6804222 31547011

[3] Chen HW, Su SF, Chien CT, Lin WH, Yu SL, Chou CC (2006). Titanium dioxide nanoparticles induce emphysema-like lung injury in mice. FASEB J.

[4] Danielsen PH, Knudsen KB, Štrancar J, Umek P, Koklič T, Garvas M (2020). Effects of physicochemical properties of TiO2 nanomaterials for pulmonary inflammation, acute phase response and alveolar proteinosis in intratracheally exposed mice. Toxicol Appl Pharmacol.

[5] Dréno B, Alexis A, Chuberre B, Marinovich M (2019). Safety of titanium dioxide nanoparticles in cosmetics. J Eur Acad Dermatol Venereol.

[6] Coelho SG, Patri AK, Wokovich AM, Chem BS, McNeil SE, Howard PC (2016). Repetitive application of sunscreen containing titanium dioxide nanoparticles on human skin. JAMA Dermatol.

[7] Naess EM, Hofgaard A, Skaug V, Gulbrandsen M, Danielsen TE, Grahnstedt S (2016). Titanium dioxide nanoparticles in sunscreen penetrate the skin into viable layers of the epidermis: a clinical approach. Photodermatol Photoimmunol Photomed.

[8] Sanders K, Degn LL, Mundy WR, Zucker RM, Dreher K, Zhao B (2012). In vitro phototoxicity and hazard identification of nano-scale titanium dioxide. Toxicol Appl Pharmacol.

[9] Jin SE, Kim EJ, Kim H, Kim H, Hwang W, Hong SW (2020). In vitro and in vivo toxicological evaluation of transition metal-doped titanium dioxide nanoparticles: nickel and platinum. Mater Sci Eng C Mater Biol Appl.

[10] Wu Q, Guo D, Du Y, Liu D, Wang D, Bi H (2014). UVB irradiation enhances TiO2 nanoparticle-induced disruption of calcium homeostasis in human lens epithelial cells. Photochem Photobiol.

[11] Eom Y, Song JS, Lee DY, Kim MK, Kang BR, Heo JH (2016). Effect of titanium dioxide nanoparticle exposure on the ocular surface: an animal study. Ocul Surf.

[12] Han JY, Kang B, Eom Y, Kim HM, Song JS (2017). Comparing the effects of particulate matter on the ocular surfaces of normal eyes and a dry eye rat model. Cornea..

[13] Jo DH, Kim JH, Son JG, Song NW, Kim YI, Yu YS (2014). Anti-angiogenic effect of bare titanium dioxide nanoparticles on pathologic neovascularization without unbearable toxicity. Nanomedicine..

[14] del Amo EM, Rimpelä A, Heikkinen E, Kari OK, Ramsay E, Lajunen T (2017). Pharmacokinetic aspects of retinal drug delivery. Prog Retin Eye Res.

[15] Frey T, Antonetti DA (2011). Alterations to the blood-retinal barrier in diabetes: cytokines and reactive oxygen species. Antioxid Redox Signal.

[16] Díaz-Coránguez M, Ramos C, Antonetti DA (2017). The inner BRB: cellular basis and development. Vis Res.

[17] van der Wijk AE, Vogels IMC, van Noorden CJF, Klaassen I, Schlingemann RO (2017). TNFα-induced disruption of the blood-retinal barrier in vitro is regulated by intracellular 3′,5′-cyclic adenosine monophosphate levels. Invest Ophthalmol Vis Sci.

[18] Tsukita S, Tanaka H, Tamura A (2019). The claudins: from tight junctions to biological systems. Trends Biochem Sci.

[19] Lv J, Hu W, Yang Z, Li T, Jiang S, Ma Z (2018). Focusing on claudin-5: a promising candidate in the regulation of BBB to treat ischemic stroke. Prog Neurobiol.

[20] Garcia MA, Nelson WJ, Chavez N. Cell-cell junctions organize structural and signaling networks. Cold Spring Harb Perspect Biol. 2018:10. 10.1101/cshperspect.a029181 PMC5773398 28600395

[21] Inai T, Kobayashi J, Shibata Y (1999). Claudin-1 contributes to the epithelial barrier function in MDCK cells. Eur J Cell Biol.

[22] Brown RC, Morris AP, O’Neil RG (2007). Tight junction protein expression and barrier properties of immortalized mouse brain microvessel endothelial cells. Brain Res.

[23] Ma SC, Li Q, Peng JY, Zhouwen JL, Diao JF, Niu JX (2017). Claudin-5 regulates blood-brain barrier permeability by modifying brain microvascular endothelial cell proliferation, migration, and adhesion to prevent lung cancer metastasis. CNS Neurosci Ther.

[24] Kaplan HJ, Chiang CW, Chen J, Song SK (2010). Vitreous volume of the mouse measured by quantitative high-resolution MRI. Investig Ophthalmol Vis Sci.

[25] Kaufmann F, Lacoste C (1986). Vitreous fluorescein accumulation determined by in vivo fluorophotometry and by vitreous extraction in normal and diabetic rats. Diabetologia..

[26] Jeon SK, Kim EJ, Lee J, Lee S (2016). Potential risks of TiO2 and ZnO nanoparticles released from sunscreens into outdoor swimming pools. J Hazard Mater.

[27] Venkatesan AK, Reed RB, Lee S, Bi X, Hanigan D, Yang Y (2018). Detection and sizing of Ti-containing particles in recreational waters using single particle ICP-MS. Bull Environ Contam Toxicol.

[28] David Holbrook R, Motabar D, Quinones O, Stanford B, Vanderford B, Moss D (2013). Titanium distribution in swimming pool water is dominated by dissolved species. Environ Pollut.

[29] Weng Y, Liu J, Jin S, Guo W, Liang X, Hu Z (2017). Nanotechnology-based strategies for treatment of ocular disease. Acta Pharm Sin B.

[30] Mittal N, Kaur G (2019). Investigations on polymeric nanoparticles for ocular delivery. Adv Polymer Techn..

[31] De Matteis V, Rizzello L. Noble metals and soft bio-Inspired nanoparticles in retinal diseases treatment: a perspective. Cells. 2020:9. 10.3390/cells9030679 PMC7140625 32164376

[32] Tahara K, Karasawa K, Onodera R, Takeuchi H (2017). Feasibility of drug delivery to the eye's posterior segment by topical instillation of PLGA nanoparticles. Asian J Pharm Sci.

[33] Chetoni P, Burgalassi S, Monti D, Tampucci S, Tullio V, Cuffini AM (2016). Solid lipid nanoparticles as promising tool for intraocular tobramycin delivery: pharmacokinetic studies on rabbits. Eur J Pharm Biopharm.

[34] Mohammadpour M, Hashemi H, Jabbarvand M, Delrish E (2014). Penetration of silicate nanoparticles into the corneal stroma and intraocular fluids. Cornea..

[35] Xu Q, Boylan NJ, Suk JS, Wang YY, Nance EA, Yang JC (2013). Nanoparticle diffusion in, and microrheology of, the bovine vitreous ex vivo. J Control Release.

[36] Wong LL, Barkam S, Seal S, McGinnis JF (2019). Temporal distribution patterns of alexa fluor 647-conjugated CeNPs in the mouse retina after a single intravitreal injection. Adv Exp Med Biol.

[37] Koo H, Moon H, Han H, Na JH, Huh MS, Park JH (2012). The movement of self-assembled amphiphilic polymeric nanoparticles in the vitreous and retina after intravitreal injection. Biomaterials..

[38] Eriksen AZ, Brewer J, Andresen TL, Urquhart AJ (2017). The diffusion dynamics of PEGylated liposomes in the intact vitreous of the ex vivo porcine eye: a fluorescence correlation spectroscopy and biodistribution study. Int J Pharm.

[39] Melgar-Asensio I, Kandela I, Aird F, Darjatmoko SR, de Los RC, Sorenson CM (2018). Extended intravitreal rabbit eye residence of nanoparticles conjugated with cationic arginine peptides for intraocular drug delivery: in vivo imaging. Invest Ophthalmol Vis Sci.

[40] Strobel C, Torrano AA, Herrmann R, Malissek M, Bräuchle C, Reller A (2014). Effects of the physicochemical properties of titanium dioxide nanoparticles, commonly used as sun protection agents, on microvascular endothelial cells. J Nanopart Res.

[41] Black RA, Rauch CT, Kozlosky CJ, Peschon JJ, Slack JL, Wolfson MF (1997). A metalloproteinase disintegrin that releases tumour-necrosis factor-alpha from cells. Nature..

[42] Yang J, LeBlanc ME, Cano I, Saez-Torres KL, Saint-Geniez M, Ng YS (2020). ADAM10 and ADAM17 proteases mediate proinflammatory cytokine-induced and constitutive cleavage of endomucin from the endothelial surface. J Biol Chem..

[43] Yang WS, Kim JJ, Lee MJ, Lee EK, Park SK (2018). ADAM17-mediated ectodomain shedding of toll-like receptor 4 as a negative feedback regulation in lipopolysaccharide-activated aortic endothelial cells. Cell Physiol Biochem.

[44] Garton KJ, Gough PJ, Philalay J, Wille PT, Blobel CP, Whitehead RH (2003). Stimulated shedding of vascular cell adhesion molecule 1 (VCAM-1) is mediated by tumor necrosis factor-alpha-converting enzyme (ADAM 17). J Biol Chem.

[45] Tsakadze NL, Sithu SD, Sen U, English WR, Murphy G, D'Souza SE (2006). Tumor necrosis factor-alpha-converting enzyme (TACE/ADAM-17) mediates the ectodomain cleavage of intercellular adhesion molecule-1 (ICAM-1). J Biol Chem.

[46] Koenen RR, Pruessmeyer J, Soehnlein O, Fraemohs L, Zernecke A, Schwarz N (2009). Regulated release and functional modulation of junctional adhesion molecule a by disintegrin metalloproteinases. Blood..

[47] Cui D, Arima M, Takubo K, Kimura T, Horiuchi K, Minagawa T (2015). ADAM12 and ADAM17 are essential molecules for hypoxia-induced impairment of neural vascular barrier function. Sci Rep.

[48] Dey M, Baldys A, Sumter DB, Göoz P, Luttrell LM, Raymond JR (2010). Bradykinin decreases podocyte permeability through ADAM17-dependent epidermal growth factor receptor activation and zonula occludens-1 rearrangement. J Pharmacol Exp Ther.

[49] Shen M, Hu M, Fedak PWM, Oudit GY, Kassiri Z (2018). Cell-specific functions of ADAM17 regulate the progression of thoracic aortic aneurysm. Circ Res.

[50] Cristian RE, Mohammad IJ, Mernea M, Sbarcea BG, Trica B, Stan MS, et al. Analyzing the interaction between two different types of nanoparticles and serum albumin. Materials (Basel). 2019:12. 10.3390/ma12193183 PMC6804176 31569341

[51] Whitwell H, Mackay RM, Elgy C, Morgan C, Griffiths M, Clark H (2016). Nanoparticles in the lung and their protein corona: the few proteins that count. Nanotoxicology..

[52] Urbančič I, Garvas M, Kokot B, Majaron H, Umek P, Cassidy H (2018). Nanoparticles an wrap epithelial cell membranes and relocate them across the epithelial cell layer. Nano Lett.

[53] Sel S, Kalinski T, Enssen I, Kaiser M, Nass N, Trau S (2012). Expression analysis of ADAM17 during mouse eye development. Ann Anat.

[54] Li CH, Shyu MK, Jhan C, Cheng YW, Tsai CH, Liu CW (2015). Gold nanoparticles increase endothelial paracellular permeability by altering components of endothelial tight junctions, and increase blood-brain barrier permeability in mice. Toxicol Sci.

[55] Liao PL, Lin CH, Li CH, Tsai CH, Ho JD, Chiou GC (2017). Anti-inflammatory properties of shikonin contribute to improved early-stage diabetic retinopathy. Sci Rep.

