# Supplementary material for: Titanium dioxide nanoparticles impair the inner blood-retinal barrier and retinal electrophysiology through rapid ADAM17 activation and claudin-5 degradation
Source: Part Fibre Toxicol. 2021 Jan 9;18:4. doi: 10.1186/s12989-020-00395-7 (PMC7796566; doi:10.1186/s12989-020-00395-7)
Supplement: Supplementary file 1 — Additional file 1: Table S1. Characteristics of TiO2 particles. Table S2. Effects of TiO2 particles on cell viability (MTT assay). Table S3. Observations on mice intraocular pressure (IOP) of intravitreal treatment with TiO2-NP. [file 12989_2020_395_MOESM1_ESM.docx]

**Supplemental Figures**

**Figure S1.**

**
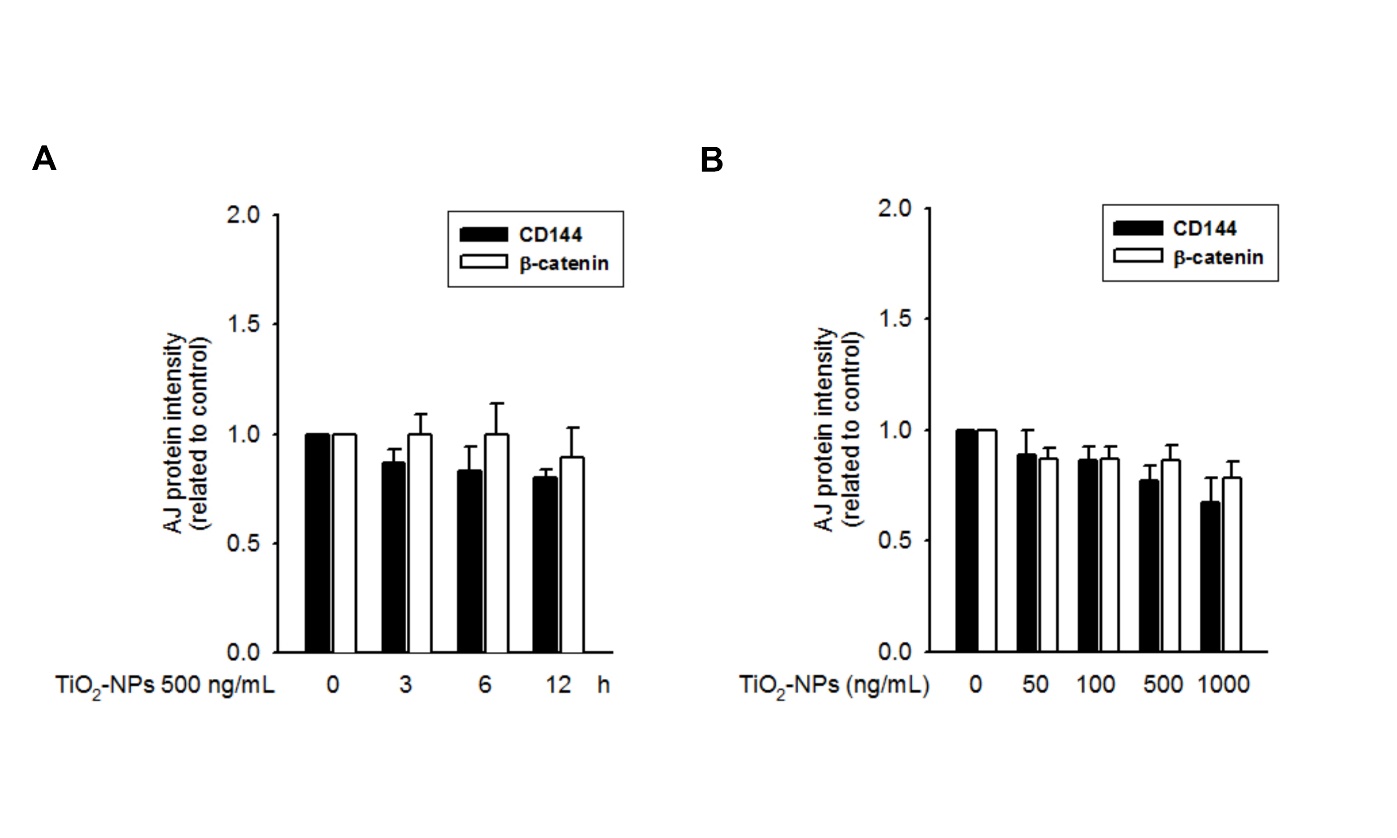
**

**Figure S1.** **TiO_2_-NPs did not affect the expression of AJ proteins.** The quantification histograms showed no significant changes in the protein expression level of AJ members (CD144 and β-catenin) in TiO_2_-NP-treated bEnd.3 cells. The representative images were presented in Figure 1. (A) time-dependent treatment. (B) mass-dependent treatment.

**Figure S2.**

**
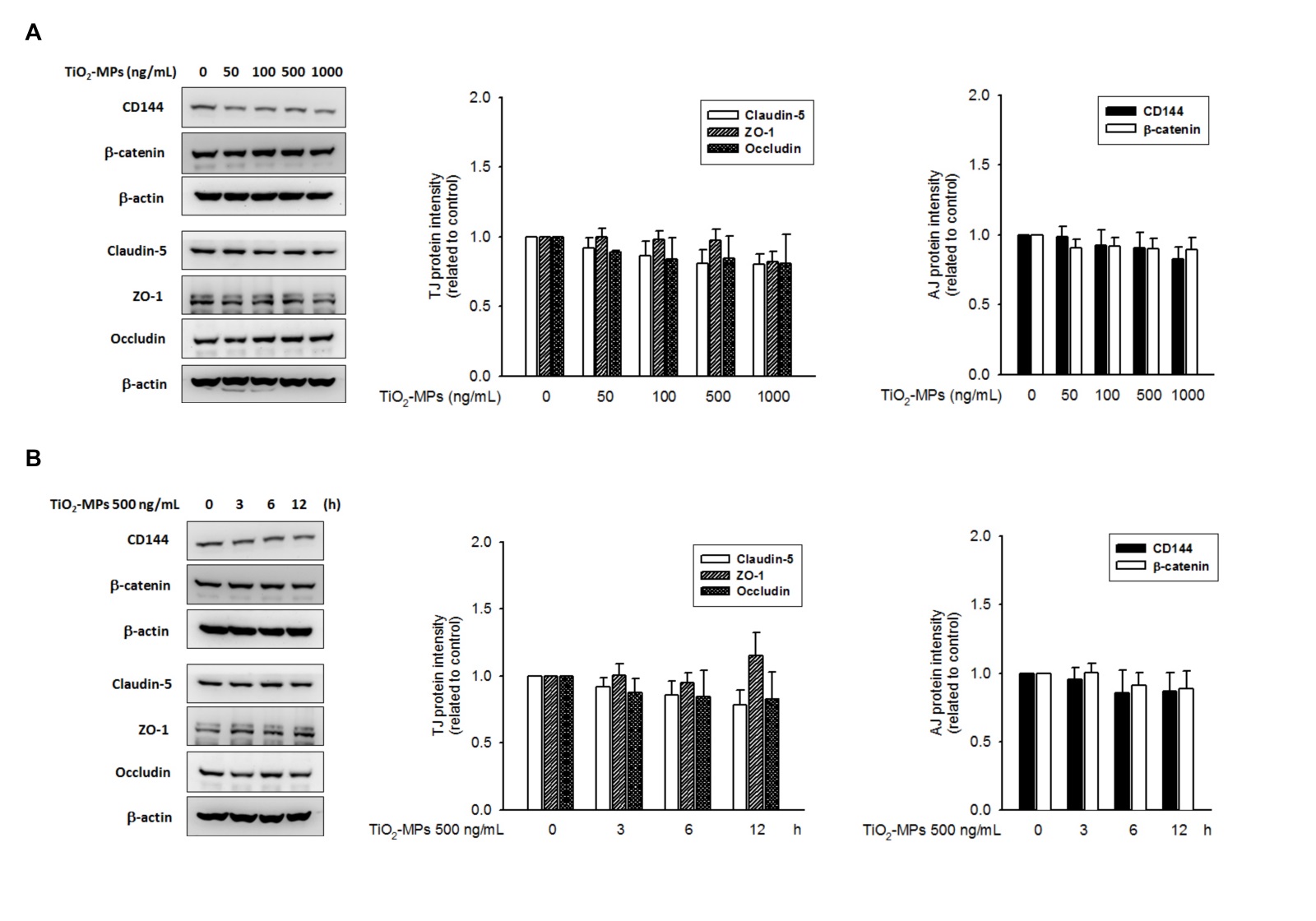
**

**Figure S2.** **TiO_2_-MPs did not affect the expression of TJ/AJ proteins.** After treatment with TiO_2_-MPs, there were no significant changes in the protein expression level of TJ members (claudin-5, ZO-1 and occludin) and AJ members (CD144 and β-catenin) in treated bEnd.3 cells. (A) mass-dependent treatment. (B) time-dependent treatment.

**Figure S3.**


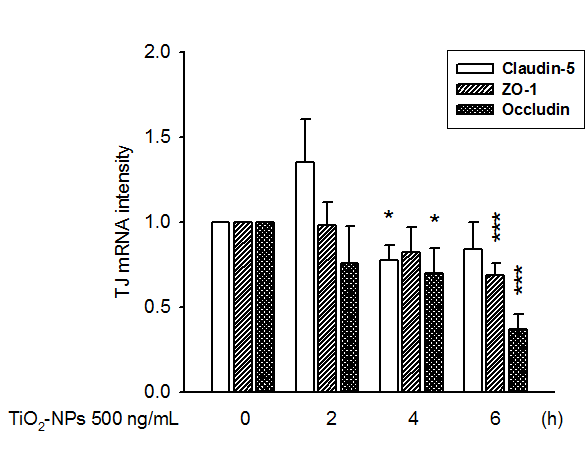


**Figure S3. Changes in mRNA level of claudin-5, ZO-1 and occludin in TiO_2_-NP-treated bEnd.3 cells.** Following 2-6 h incubation in the presence of 500 ng/ml TiO_2_-NPs, total RNA was isolated, and the first strand cDNA was made by MMLV reverse transcriptase. The mRNA quantification was conducted by the SYBR green system employing a LightCycler^®^Nano instrument. Relative expression of transcripts was analyzed by the ΔΔCq method with normalization to the level of housekeeping gene, GAPDH. We found that the mRNA level of ZO-1 and occludin was down-regulated after 4-6 h TiO_2_-NP treatment. However, the mRNA expressing level of claudin-5 was without significant changes. The primer sets for qPCR: ZO-1 (forward 5’- CACCTTTTGATAATCAGCACTC-3’, reverse 5’- CTCTAGGTGCCT GTTCGTAACG-3’); occludin (forward 5’- TCAGGGAATATCCACCTATCACTTC -3’, reverse 5’- CATCAGCAGCAGCCATGT ACTCTTC -3’); claudin-5 (forward 5’- TTCGCCAACATTGTCGTCC -3’, reverse 5’- TCTTCTTGTCGTAGTCGCCG -3’); β-actin (forward 5’- TGTCCACCTTCCAGCAGATGT -3’, reverse 5’- AGCTCAG TAACAGTCCGCCTAG -3’). *p < 0.05, ***p < 0.001, indicates statistically significant difference from the control treatment.

**Figure S4.**

**
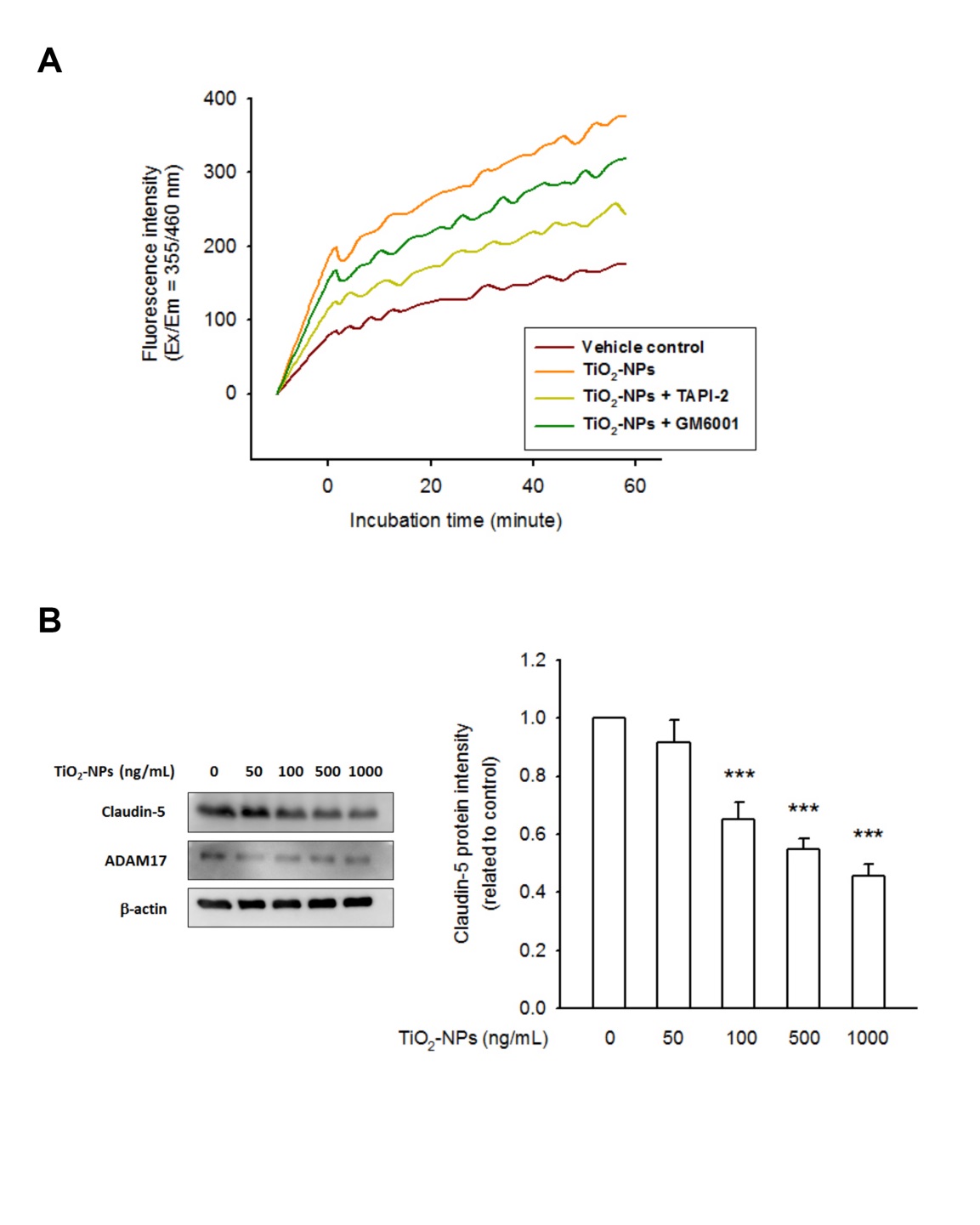
**

**Figure S4.** **TiO_2_-NP activated ADAM17 directly, contributing to rapid claudin-5 protein degradation.** (A) ADAM17 kinetics of bEnd.3 cell lysate was performed as described in Materials and Methods. The basal ADAM17 activity (vehicle control) was showed in brown curve. In combination with TiO_2_-NP (500 ng/mL), an increasing fluorescence illustrated the induction of ADAM17 activity by TiO_2_-NP (orange curve), whereas the fluorescence signals were prevented by GM6001 (dark green curve) and TAPI-2 (light green curve). (B) bEnd.3 cell lysate was prepared and aliquoted (20 μg protein/vial), then incubated with TiO_2_-NPs (50-1000 ng/mL) at 37℃ for 3 h. Another vial, without TiO2-NP treatment, was used as control. Next, the reaction was stopped by the addition of SDS-PAGE sample loading buffer. The amount of claudin-5 was detected by immunoblotting. Data showed an apparent protein degradation of claudin-5, whereas ADAM17 protein level was not changed, whatever the presence or absence of TiO2-NP treatment. (***p < 0.001 indicates statistically significant difference from the control group).

**Figure S5.**


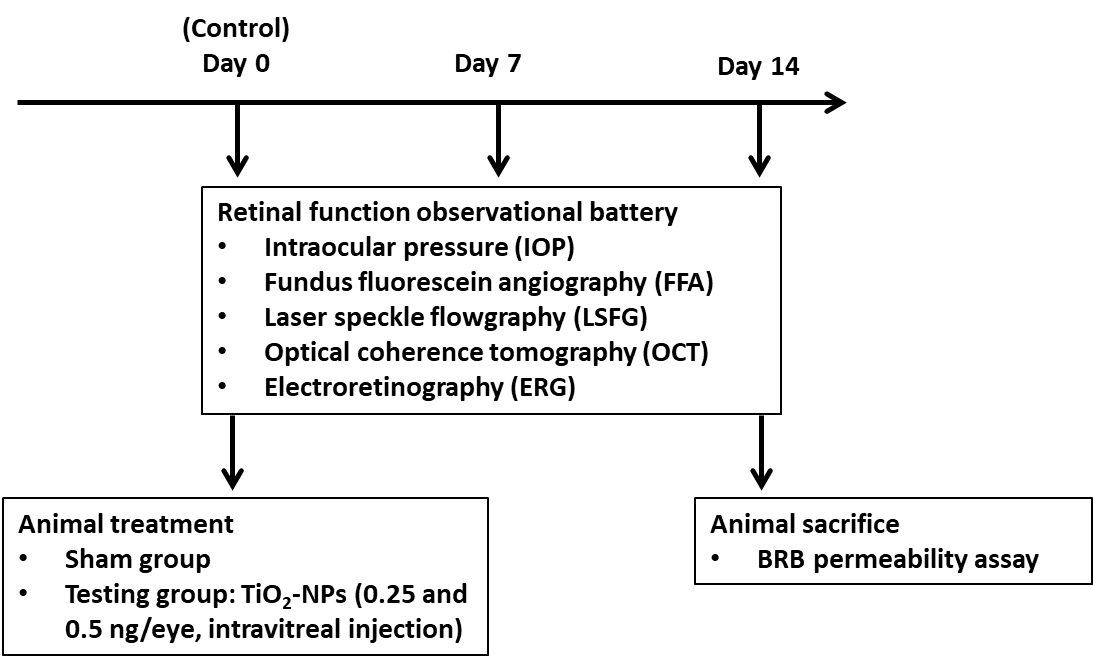


**Figure S5. Scheme of animal treatment.** On day 0, the retinal function observational battery, including intraocular pressure (IOP), fundus photography (FP), fundus fluorescein angiography (FFA), laser speckle flowgraphy (LSFG), optical coherence tomography (OCT), and electroretinogram (ERG), was performed in all animals, and the results represented the control status. For each test, the apparatus and operating instruction was described in Materials and Methods. Next, the mice in the testing groups received a single-dose, ITV injection of TiO_2_-NPs (0.25 and 0.5 ng/eye) in their right eye, whereas a vehicle injection was performed in the sham group. Briefly, mice were anesthetized, and ITV injection was fulfilled by using Hamilton microsyringe equipped with 30G needles. The injected volume was 1 μL per eye. At day 7 and 14 post dosing, retinal function was re-evaluated as described earlier. At the end of the study, the eyeballs were dissected for additional examination.

**Figure S6.**

**
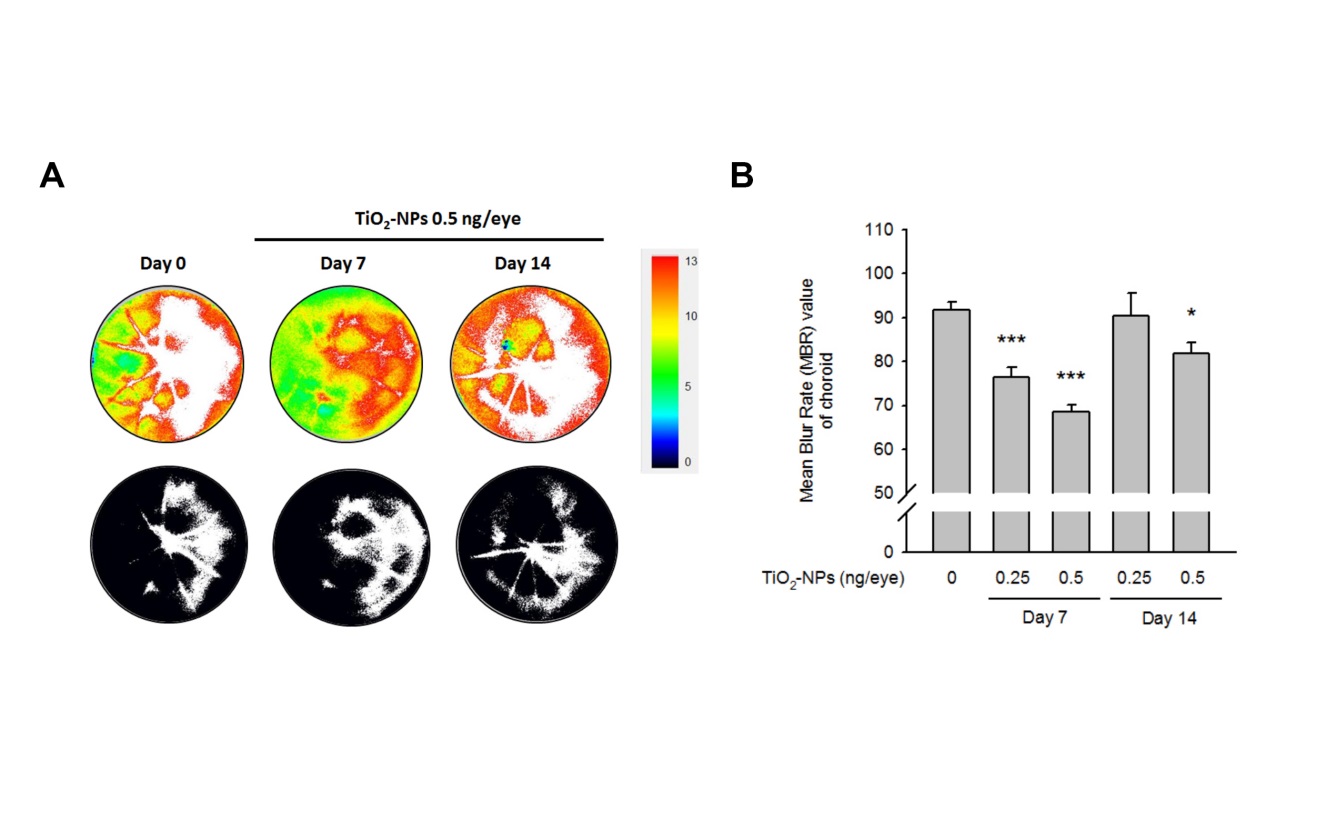
**

**Figure S6**. **Reduction of choroid blood flow was evidenced in TiO_2_-NP-treated mice.** (A) Figure of the MBR color-coded maps showed an obvious reduction of choroid blood flow on day 7 post dosing. (B) MBR values of choroid were significantly reduced in TiO_2_-NP-treated groups at day 7, with a dose relationship. The recovery of MBR values was at day 14. *p < 0.05, and ***p < 0.001 indicates statistically significant difference from the control group).

**Figure S7.**

**
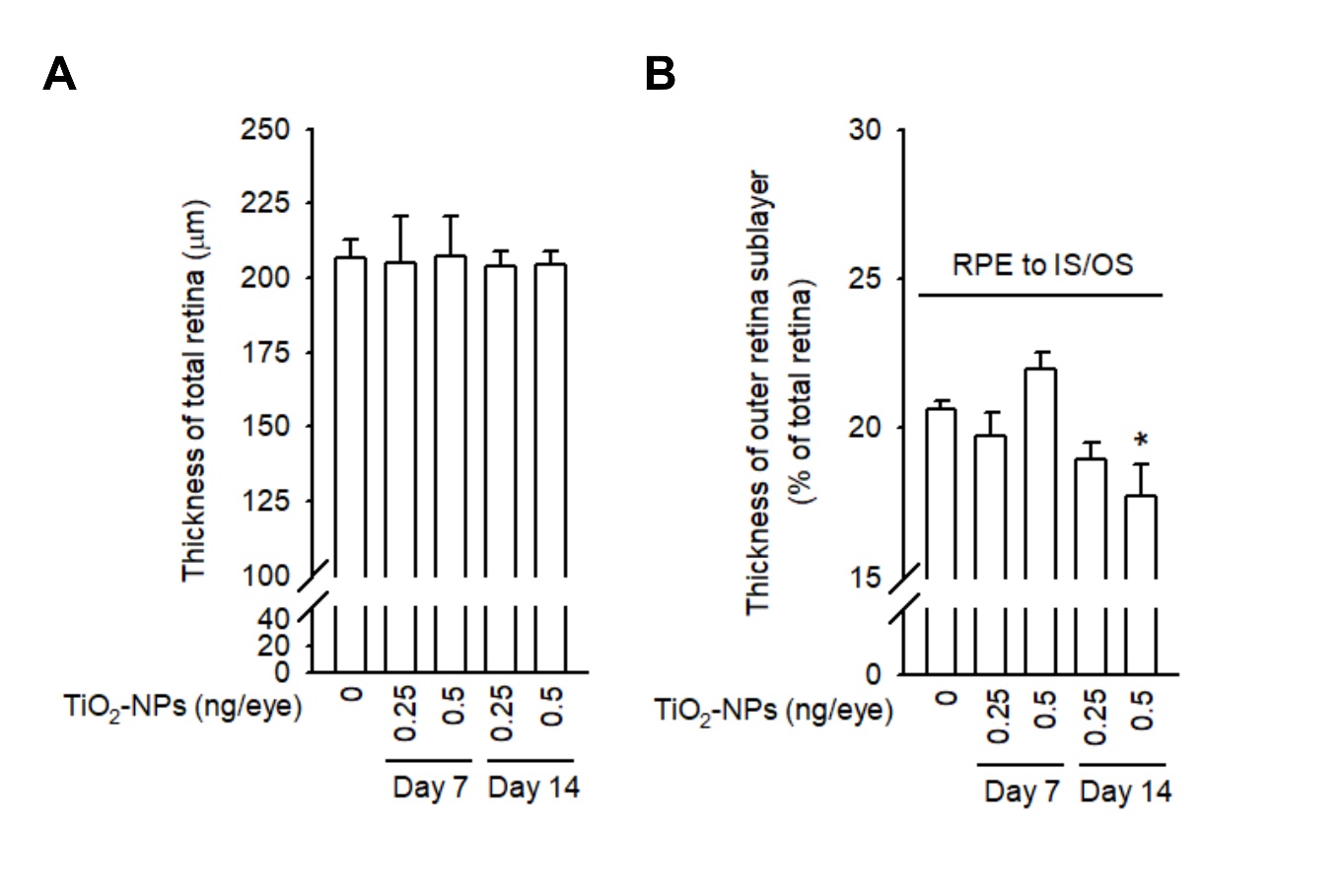
**

**Figure S7.** **Intravitreal exposure of TiO_2_-NPs changed the thickness of retinal sublayers.** Representative SD-OCT scan images were showed in Figure 7. (A) The thickness of the total retina was automatically computed by software. Neither the retina structure nor the total retina thickness was found changed at day 7 and 14 after TiO_2_-NP treatment. (B) An inappreciable reduction in the outer sublayer, comprises the inner and outer segments (IS/OS) of photoreceptors, was computed at day 14 (17.71 ± 1.08% of total retina thickness, 0.5 ng/eye treated group) compared to that on day 0 (20.61 ± 0.31%). *p < 0.05, indicates statistically significant difference from the control (day 0).
