# Supplementary material for: Titanium dioxide nanoparticles impair the inner blood-retinal barrier and retinal electrophysiology through rapid ADAM17 activation and claudin-5 degradation
Source: Part Fibre Toxicol. 2021 Jan 9;18:4. doi: 10.1186/s12989-020-00395-7 (PMC7796566; doi:10.1186/s12989-020-00395-7)
Supplement: Supplementary file 2 — Additional file 2: Figure S1. TiO2-NPs did not affect the expression of AJ proteins. Figure S2. TiO2-MPs did not affect the expression of TJ/AJ proteins. Figure S3. Changes in mRNA level of claudin-5, ZO-1 and occludin in TiO22-NP-treated bEnd.3 cells. Figure S4. TiO2-NP activated ADAM17 directly, contributing to rapid claudin-5 protein degradation. Figure S5. Scheme of animal treatment. Figure S6. Reduction of choroid blood flow was evidenced in TiO2-NP-treated mice. Figure S7. Intravitreal exposure of TiO2-NPs changed the thickness of retinal sublayers. [file 12989_2020_395_MOESM2_ESM.docx]

**Table S1. Characteristics of TiO_2_ particles.**

|  | **Characterization of TiO_2_ particles** | |
| --- | --- | --- |
|  | **TiO_2_-NPs** | **TiO_2_-MPs** |
| **TEM image** | 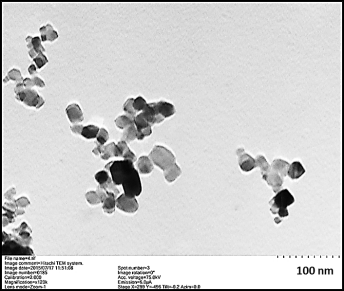 | 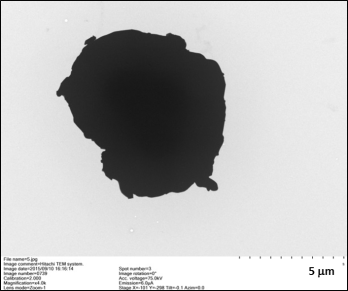 |
| **Primary particle size (TEM)** | 42 ± 3 nm | 4655 ± 371 nm |
| **Hydrodynamic diameter (DLS)** | 184.7 ± 64.1 nm | n.d. |
| **Zeta potential (mV)** | −14.17 ± 7.42 | −11.80 ± 2.76 |
| **UV-Vis absorption spectrum** | 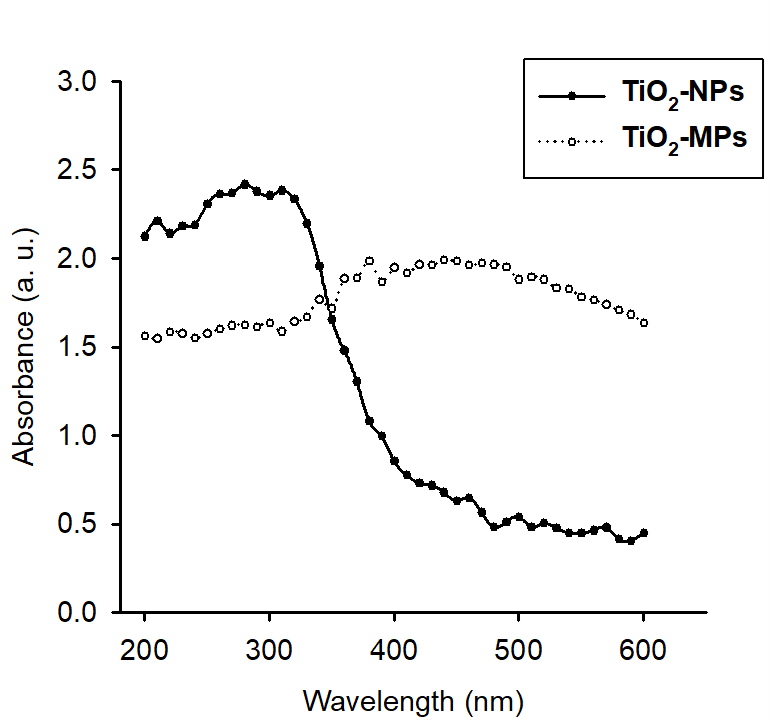 | |

Notes:

1. TiO_2_ samples (1 ppb) were placed on a Cu mesh grid, and lyophilized overnight at room temperature. The shape and size of TiO_2_ particles were observed by transmission electron microscopy (TEM; Hitach HT-7700, Japan), operating at 200 kV. TEM revealed that the TiO_2_-NPs were polydisperse, with an average diameter of 42 ± 3 nm.
2. Hydrodynamic diameter was measured by using dynamic light scattering (DLS) method. TiO_2_-NPs suspension (1 ppb) was prepared in culture medium (pH 7.4) freshly, and a brief sonication is necessary before testing using NanoSight NS300 (Malvern Panalytical Ltd, Malvern, UK). Measurements were taken 5-6 times and the mean hydrodynamic diameter was automatically calculated by the equipped software. Measurements of hydrodynamic diameter were also performed in 30% glycerol solution and PBS, and the measures were 177.3 ± 76.1 and 215.5 ± 99.0 nm, respectively. The “n.d.” means “not detected” due to rapid sedimentation of TiO_2_-MPs.
3. Measurements on the electrophoretic mobility (ζ-potential) were conducted with a Zetasizer 300 device (Malvern Instruments, Worcestershire, UK). Samples were prepared as described in DLS measurement. Mean ζ-potential was obtained from at least 5 measures.
4. Optical properties of TiO_2_ were measured by subjecting the sample to UV-Vis spectrophotometer within the range 200 to 600 nm and absorbance was plotted on a graph. TiO_2_-NPs showed a better absorbance at UVB spectrum (280-320 nm), as compared to TiO_2_-MPs.
5. Data are expressed as means ± S.E.M.

**Table S2. Effects of TiO_2_ particles on cell viability (MTT assay).**

|  | **Cell viability (% related to control)** | | | | | | | | | | |
| --- | --- | --- | --- | --- | --- | --- | --- | --- | --- | --- | --- |
|  |  | **TiO_2_-NPs (ng/mL)** | | | | | **TiO_2_-MPs (ng/mL)** | | | | |
|  | **0** | **10** | **50** | **100** | **500** | **1000** | **10** | **50** | **100** | **500** | **1000** |
| **bEnd.3** | 100.0±0.0 | 95.1±1.1 | 93.7±1.3* | 90.8±1.6* | 86.0±2.3* | 83.8±2.1* | 98.6±3.5 | 97.9±4.5 | 96.5±3.5 | 94.7±3.5 | 92.8±3.7 |
| **HREC** | 100.0±0.0 | 82.0±2.6* | 77.6±3.8* | 76.6±2.7* | 76.1±2.0* | 67.9±3.2* | n.d. | n.d. | n.d. | n.d. | n.d. |
| **ARPE-19** | 100.0±0.0 | 97.2±2.9 | 96.0±2.3 | 96.5±1.0 | 93.5±4.8 | 94.2±0.5 | 98.5±1.6 | 94.8±0.3 | 93.4±1.9* | 91.6±2.0* | 89.6±0.3* |

^1^ Cell viability was evaluated by using the MTT assay. Briefly, cells were treated with series of concentrations of TiO_2_ particles for 24 h. After incubation, MTT reagent was added to the cultures, and cells were incubated at 37°C for another 2 h. Finally, the medium was gently removed, and the formazan was dissolved in DMSO. Absorption was measured at 570 nm. Data shown here were generated from at least 3 independent assays (N = 4–6). Generally, the cytotoxicity of TiO_2_ particles < 1000 ng/mL is limited in bEnd.3 cells and RPECs, whereas the primary HREC was vulnerable to TiO_2_-NP treatment.

^2^ * p < 0.05 indicates a statistically significant difference from the control group.

**Table S3. Observations on mice intraocular pressure (IOP) of intravitreal treatment with TiO_2_-NP.**

|  | Intraocular pressure (IOP, mmHg) | | |
| --- | --- | --- | --- |
| TiO_2_-NPs (ng/eye) | Day 0 | Day 7 | Day 14 |
| 0.25 | 8.60 ± 0.21 | 8.14 ± 0.40 | 8.50 ± 0.38 |
| 0.5 |  | 8.43 ± 0.43 | 7.67 ± 0.33* |

^1^ Mice were received a single-dose TiO_2_-NP as described in Materials and Methods. The IOP was measured at day 0, 7 and 14, by using tonometer (model TV02, Icare Finland Oy, Espoo, Finland). The IOP of normal mice are 8-10 mmHg. (N = 6–8).

^2^ Data were expressed as mean ± SEM. * p < 0.05, indicates a statistically significant difference from the control group.
